# A haplotype-resolved nutmeg genome reveals XY sex chromosome evolution and enables molecular sexing

**DOI:** 10.1038/s42003-026-10355-0

**Published:** 2026-05-28

**Authors:** Katarina Matvijev, Sufang Wang, Mengjun Xiao, Ashini D. Mahadura, Ray Ming, Dmitry A. Filatov

**Affiliations:** 1 Department of Biology, University of Oxford, Oxford, UK; 2 School of Life Science and Technology, Northwestern Polytechnical University, Xi’an, China; 3 Centre for Genomics and Biotechnology, Fujian Provincial Key Laboratory of Haixia Applied Plant Systems Biology, Key Laboratory of Genetics, Breeding and Multiple Utilization of Crops, Ministry of Education, Fujian Agriculture and Forestry University, Fuzhou, China; 4 Department of Botany and Zoology, Faculty of Science, Masaryk University, Brno, Czechia

**Keywords:** Evolutionary genetics, Genome evolution

## Abstract

Nutmeg (*Myristica fragrans* Houtt.) is a dioecious species that produces one of the most valuable spices and has peculiar holocentric chromosomes with diffuse centromere. The impact of holocentricity on the evolution of sex chromosomes is not well understood. Here, we present the first fully annotated haplotype-resolved chromosome-level genome of a male nutmeg plant, identify its sex chromosomes and analyse evolution of the sex-linked region (SLR). We demonstrate that nutmeg has a male heterogametic (XY) system, with a 0.23 Mb X-linked SLR and a 3.1 Mb Y-linked SLR with limited evidence for Y-degeneration. Low X:Y synonymous divergence for X- and Y-linked homologs (K_s_ < 2.5%) indicates recent evolution of sex linkage, likely through loss(es) of homologous X-linked region(s). Leveraging the new genome and SLR sequences, we developed PCR-based molecular marker for reliable sexing of nutmeg seedlings, enabling efficient management of plantations by ensuring the optimal male to female ratio (1:10).

## Abstract (web summary)

The analysis of genomic differences between male and female nutmeg plants identified the sex-determining region, revealed sex chromosome evolution in nutmegs and yielded a molecular marker to identify males and females in nutmeg seedlings.

## Introduction

The evolution of sex chromosomes in plants is a persistently fascinating topic. Most animals have separate sexes, and sex chromosomes usually evolve and re-evolve via consecutive turnovers^1^. On the other hand, in flowering plants, separate sexes (dioecy) are uncommon (ca. 6%^2^) and sex chromosomes often evolve de novo during or after transition to dioecy, though turnovers in ancestrally dioecious angiosperm clades, such as willows, have also been described^3^. Even though significant advances in understanding of sex chromosome evolution have been made, the origins and dynamics of plants' sex chromosomes are still not fully understood^4^. The majority of dioecious angiosperms with an identified reproductive system have male-heterogamety (XX/XY sex chromosomes in females and males, respectively), although female-heterogamety (ZZ/ZW) has also been reported^5^. Sex chromosomes that can be clearly distinguished under a microscope (heteromorphic) were described only in a few plants, such as *Silene*^6^ and *Rumex*^7^, with most dioecious plants having morphologically indistinguishable (homomorphic) sex chromosomes^8^. In heteromorphic sex chromosomes, the sex-linked region (SLR) usually spans most of the chromosome length, whereas in homomorphic chromosomes, the SLR is usually smaller^9^. Sex chromosomes in plants were previously considered to be of a recent origin due to their tendency to be homomorphic, but the accumulation of genome sequence data revealed the existence of species with old homomorphic sex chromosomes, such as ginkgo^10^. Although dioecy occurs in low numbers in most orders of angiosperms, certain families are exclusively or almost exclusively dioecious^11,12^, suggesting that sex chromosomes in these families are old. However, turnovers of sex chromosomes can keep them ‘young’ even in the families where dioecy is ‘old’, such as reported for willows^3,13^. Previously, dioecy has been considered an 'evolutionary dead-end' mainly due to its rarity in angiosperms^14^ or because dioecious lineages have higher extinction rates^15^. Now it is accepted that dioecy is relatively rare, most likely due to frequent reversals from dioecy to cosexuality^11^, and that plant reproductive systems are much more evolutionary dynamic compared to animals^16^.

Highly evolutionary dynamic mating systems in plants provided an opportunity to study the early stages of de novo sex chromosome evolution when a cosexual species evolves separate sexes^4,17^. Early genetic work in the dioecious white campion revealed the presence of two sex-determining factors on the Y chromosome—the gynoecium suppression and stamen promotion factors^14^, which inspired the development of the classic two-factor model of sex chromosome evolution^18^. The analysis of sex determination in a range of species^19^ supported the two- factor model. More recently^20^, it was argued that a single-factor model accounts for sex determination in persimmon^21^ and an artificial switch to dioecy in melon^22^ illustrated how a single-factor sex determination could arise. Currently, it is widely accepted that the emergence of sex chromosomes in plants requires at least two mutations—male and female sterility mutations^23^, which can be genetic variation at a single locus (single-factor determination^20^) or at two linked loci (two-factor determination^18^). The power of the two-factor model is in its ability to explain the cause of initial recombination suppression between the sex-determining loci- linking male-promoting and female-suppressing factors prevents the formation of sterile plants that inherited both male and female sterility mutations^18^. This nascent non-recombining Y (NRY = MSY, male-specific Y) region around the sex-determining loci has a tendency to expand over time, forming evolutionary strata—the regions with distinct X:Y divergence, which was first described in humans^24^, but turned out to be a general property of sex chromosomes in animals^25^. Plant sex chromosomes typically contain small NRY^4^, with expansions reported only for a few plants with large NRY, such as in *Silene latifolia*^26–28^. The causes of NRY expansion are not fully understood, but are thought to be at least partly driven by sexually antagonistic genes^12,29,30^.

Here we report identification and analysis of homomorphic sex chromosomes in the dioecious nutmeg (*Myristica fragrans* Houtt.). Species in the nutmeg family (Myristicaceae) are generally dioecious, with 15 out of 20 genera having either exclusively or predominantly dioecious species^2^, indicating that dioecy is ancestral in this family. However, it is unclear whether sex chromosomes, if any, are old and shared between dioecious species in this family. True nutmeg, *M. fragrans*, is a tropical tree species autochthonous to the Molluccas islands, but now present in other tropical regions due to its economic relevance in the spice industry. This species is generally described as dioecious, although there are recurrent records of monoecious individuals^31,32^, which likely represent ‘inconstant’ males or females described in many dioecious species^33^. Apart from their unisexual flowers, nutmeg males and females are indistinguishable, meaning that individuals can be sexed only once they reach reproductive maturity after 8–10 years, which is a problem for nutmeg growers who have to optimise sex ratio (1 male to ~10 females) on nutmeg plantations. The previously proposed supposedly sex-linked molecular markers^34^ for sexing nutmeg seedlings before planting them do not reliably distinguish males and females in our analyses (see below). Thus, apart of intrinsic scientific interest of studying sex chromosomes and their evolution, identification of the nutmeg SLR, reported in this study, is relevant for agriculture.

Apart of economic importance, dioecy and sex chromosomes, nutmeg is interesting due to its holocentric chromosomes^35^ and interplay between holocentricity and sex chromosome evolution. Holocentricity–diffuse centromere with the mitotic spindle attaching along the entire chromosome length, is relatively rare, but it evolved multiple times in a range of animals (e.g. Lepidoptera, nematodes) and plants (e.g. rushes and sedges)^36^. Holocentric species have many peculiar features, such as inverted meiosis—segregation of sister chromatids before segregation of homologues^37,38^. Another interesting feature of holocentric species is their relative robustness to chromosome fragmentation—a chromosome fragment is usually lost in monocentrics, but it usually segregates correctly in holocentrics, raising debates whether holocentricity leads to evolution of more numerous smaller chromosomes^39^ and whether chromosome speciation is more common in holocentric than in monocentric species^40^. Distribution of recombination along the chromosomes differs between mono- and holocentric species. In monocentrics, recombination is usually suppressed around the centromere^41^, resulting in the pericentromeric region accumulating transposable elements and other repetitive DNA and losing functional genes, not dissimilar to non-recombining Y-chromosomes^42^. Accumulation of junk DNA in the pericentromeric region may lead to its expansion, and it was recently reported that pericentromeric expansion likely played a significant role in sex chromosome evolution in *Silene latifolia*^27^. Pericentromeric recombination suppression was also reported to play a significant role in initial recombination suppression on nascent plant sex chromosomes^43^. Holocentric species lack localised centromere and do not have pericentromeric recombination suppression^44^, which likely has implications for sex chromosome evolution. A combination of dioecy, sex chromosomes and holocentricity is rare in plants and nutmeg provides an opportunity to study the evolution of homomorphic sex chromosomes in holocentric plants.

Here, we first present the first fully annotated haplotype-resolved chromosome-level assembly of a male nutmeg genome. We then identify the sex-linked region using multiple approaches and analyse the features of sex-linked and adjacent regions to infer the evolution of sex chromosomes in nutmegs. Our results confirm the previous reports that nutmeg is a species with 22 homomorphic chromosomes^31,34^, but contrary to previous suggestions of female heterogamety (ZW)^34^, we report that male nutmeg plants have XY and females have XX sex chromosomes. We report the evidence for the recent expansion of nutmeg NRY and analyse evolutionary genetic processes in the old and recently added sex-linked regions. The sequence of the nutmeg Y chromosome allowed us to develop molecular markers for reliable sexing of nutmeg seedlings to optimise the sex ratio of nutmeg plantations without the need to wait ~8–10 years until flowering.

## Results

### Genome size and chromosome number

To estimate the genome size of *M. fragrans*, we used flow cytometry on three male individuals, with tomato (genome size ≈ 900 Mb) or maize B73 (genome size ≈ 2.3 Gb) as references. The genome size of *M. fragrans* was estimated to be ~590 Mb using this method (Table 1). Cytological analysis confirmed that the sex chromosomes cannot be distinguished under the microscope without specific labelling (i.e. they are homomorphic), and that the haploid number of chromosomes is *n* = 22 (Supplementary Fig. S1).

**Table 1:** Flow cytometry-based estimation of the genome size of *M. fragrans*

Nutmeg sample	Internal reference	Fluorescence intensity of internal reference	Fluorescence intensity of the nutmeg sample	Ratio	Estimate for nutmeg genome (Gb)
Male-1	Tomato	25.43	16.55	0.65	0.59
Male-1	Maize	70.27	17.48	0.25	0.57
Male-2	Tomato	24.96	16.31	0.65	0.59
Male-3	Tomato	24.79	16.32	0.66	0.59

### Genome assembly, annotation and resequencing

**Fig. 1:**
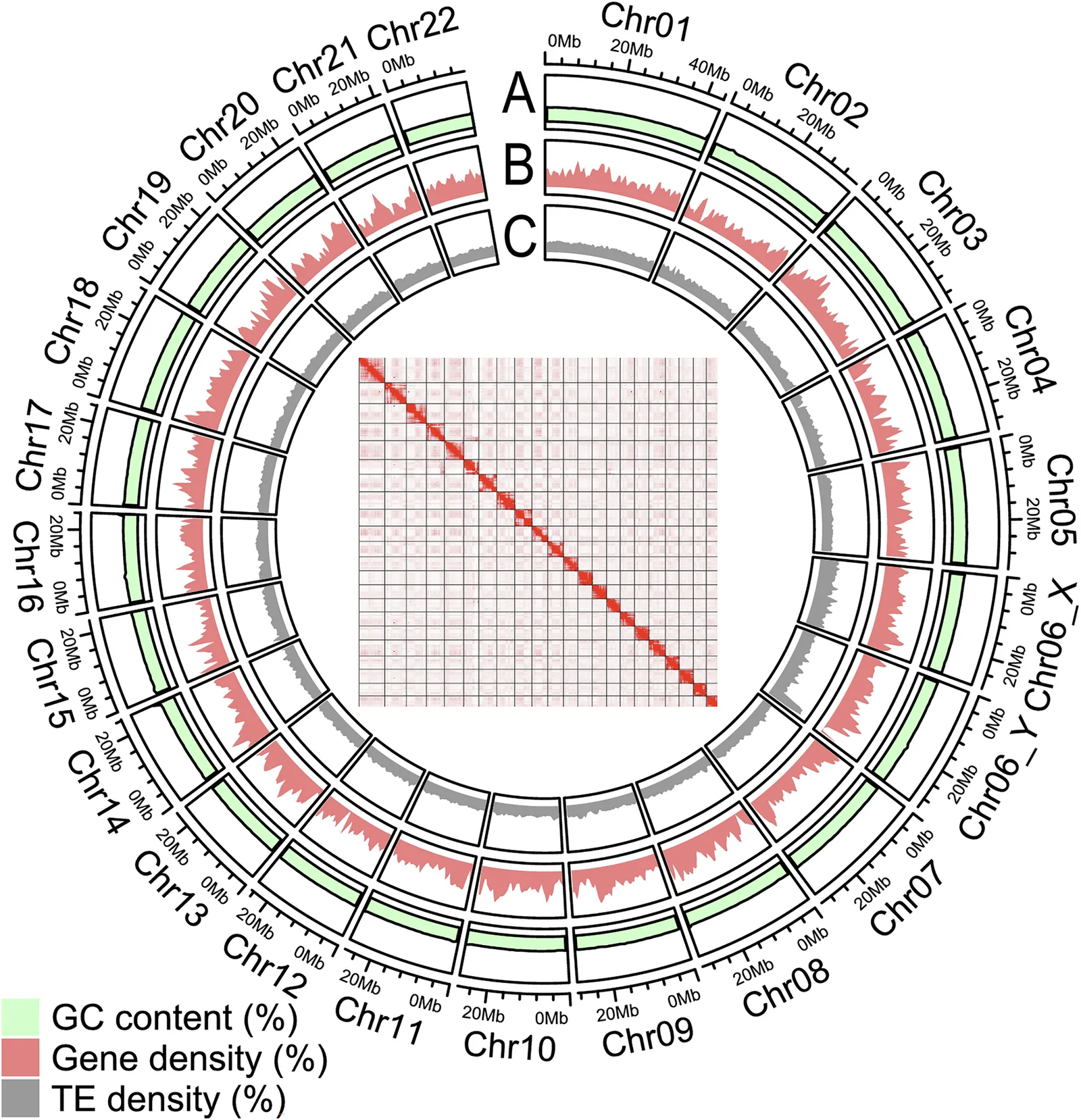
Circos plot for male genome assembly of *Myristica fragrans.* Rings indicate **A** percentage of GC content, **B** gene density and **C** TE density, all calculated at 1 Mb intervals. Hi-C heatmap of the male genome assembly is shown in the centre of the circos plot.

We generated a chromosome-level haplotype-resolved assembly of the male *M. fragrans* genome using a combination of long-read PacBio HiFi sequencing and the high-resolution chromosome conformation capture (Hi-C). We generated a total of 19,542,512,782 bp (Supplementary Table S1) of raw long read data for the reference male genome assembly, as well as more than 6 Gb of RNA-seq short read data (Supplementary Table S2). The final primary male genome assembly had a length of 641 Mb across 22 fully resolved chromosomes (Fig. 1, Table 2 and Supplementary Table S3), which is consistent with cytological chromosome count (Supplementary Fig. S1) as well as genome size estimation based on K-mers and cytometry (Table 1). Similarly, to other holocentric species^45^, the genome of *M. fragrans* has relatively low GC-content: 36.7% that is evenly distributed along the chromosomes (Fig. 1A).

**Table 2:** Summary statistics of the assembled male genome

	Primary	Haplotype 1		Haplotype 2
Scaffold length (bp)	640,827,773	633,402,135		627,476,598
Number of scaffolds	109	157		90
GC content (%)	37%	37%		37%
N50 (scaffolds)	27,097,807	27,088,625		26,872,488
Pseudochromosomes	22	22		22
Anchor rate (%)	97.61%	95.26%		95.48%
Number of gaps	5	28		22
BUSCO score	97.3%	97.3%		97.3%

Gene prediction using BRAKER3 v3.0.8^46^ identified 19,857 and 19,904 genes on haplotype 1 (hereafter 'hap1') and haplotype 2 (hereafter 'hap2'), respectively. BUSCO completeness was 97.5% and 97.2% for the two haplotypes, respectively. Transposable elements (TEs) comprised 37.42% of the hap1 and 38.60% of the hap2 sequence length (Supplementary Table S4). This relatively low TE content for a plant genome of modest size (e.g. 52% in oak^47^, 68% in tomato^48^ and 73% in *Silene conica*^49^) is possibly due to holocentricity of nutmegs. Pericentromeric regions in monocentric chromosomes are typically rarely recombining and accumulate TEs. Such regions can be very extensive in monocentric plants, with recombination occurring mainly at the ends of the chromosomes (e.g.^27,50,51^). Holocentric chromosomes lack localised centromeres and therefore do not exhibit canonical pericentromeric regions. Recombination on such chromosomes is typically evenly distributed along the chromosome length^52^. This likely prevents accumulation of TEs; e.g. in holocentric butterflies, the average TE content is similar to that in nutmeg – about 39%^53^.

Generally, genes (Fig. 1B) and TEs (Fig. 1C) were quite uniformly distributed across the genome. However, a ca. 2.5 Mb region on chromosome 6 of hap1 had an unusually high TE density and was fairly gene-poor (Fig. 1B, C), which suggested this may be a non-recombining sex-linked region. The analyses presented below confirm this hypothesis.

### Comparison with the *Myristica yunnanensis* genome

**Fig. 2:**
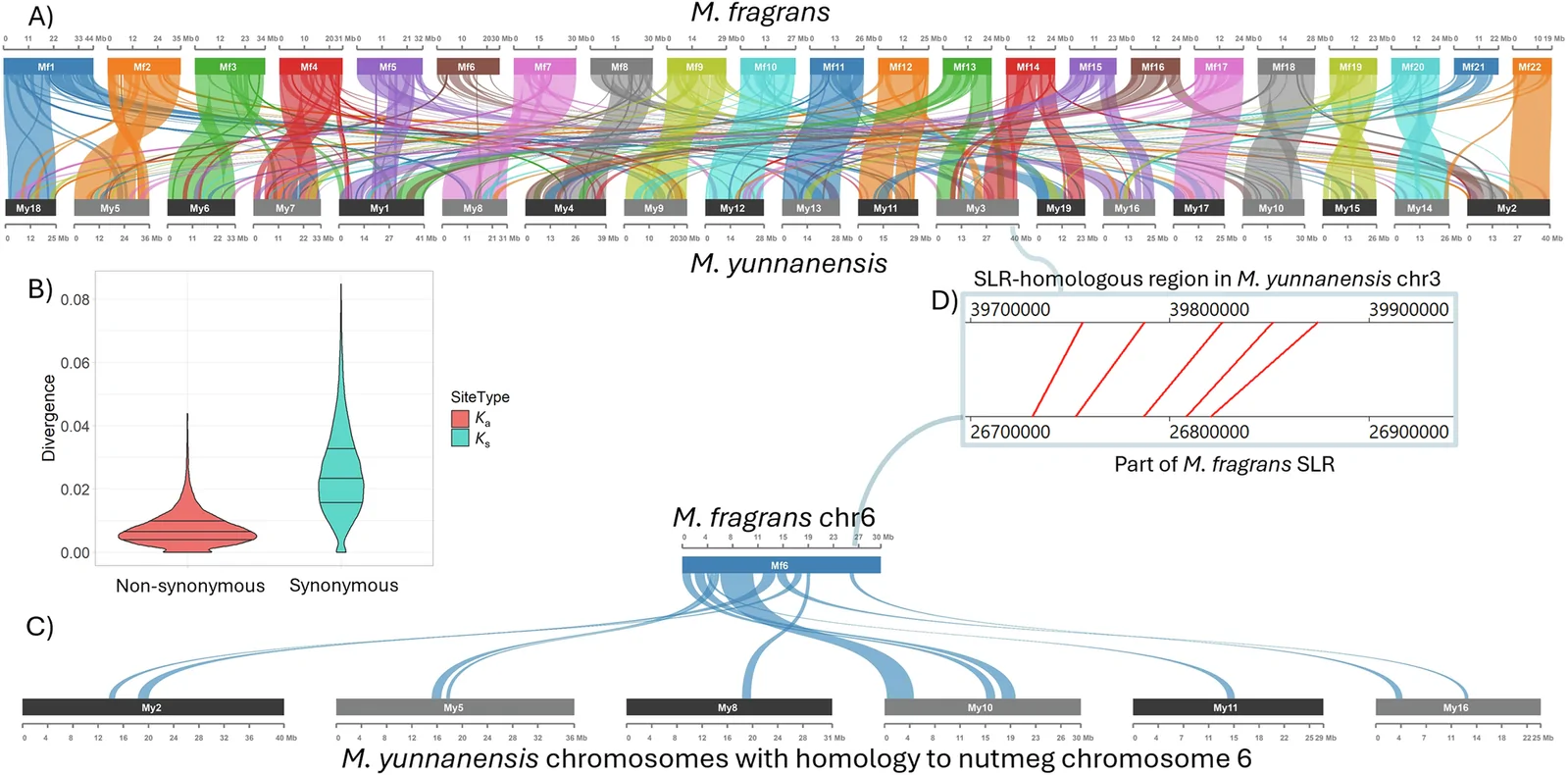
Synteny of nutmeg chromosomes with the congeneric *M. yunnanensis* genome. **A** Synteny between the two species is preserved for many chromosomes, but not for the nutmeg chromosome 6. **B** Genome-wide distribution of synonymous (*K*_s_) and non-synonymous (*K*_a_) divergence between annotated nutmeg genes and their 1-to-1 homologues in the *M. yunnanensis* genome. **C** Nutmeg chromosome 6 is fragmented in the *M. yunnanensis* genome. **D** The synteny and positions for five nutmeg sex-linked genes (from Xh1 region, see Fig. 3E) is shown with red lines.

The newly assembled nutmeg genome was compared with the recently published^54^ unphased genome of congeneric *Myristica yunnanensis*. The published *M*. *yunnanensis* genome assembly contains 19 chromosomes, and sex chromosomes were not analysed. This comparison reveals that synteny for many chromosomes is preserved, though multiple chromosome inversion, fission and fusion events are evident between *M. ynnanensis* and *M. fragrance* genomes (Fig. 2A). The preservation of synteny for many chromosomes is not too surprising given the relatively low divergence between these species: mean divergence at synonymous sites across 13,577 1-to-1 orthologs across the genome is only 2.53% (Fig. 2B), indicating recent divergence between these species. Non-synonymous divergence for the same set of genes is significantly lower than synonymous divergence (Fig. 2B), reflecting the predominance of purifying selection eliminating deleterious mutations affecting protein sequence.

Synteny analysis for the nutmeg chromosome 6 reveals many regions with micro-synteny to different *M*. *yunnanensis* chromosomes, but no clearly macro-syntenic chromosomes (Fig. 2C). Lack of macro-synteny of nutmeg chromosome 6 with *M. yunnanensis* chromosomes precludes identification of *M. yunnanensis* likely sex chromosome based on synteny analysis only. Small part of the *M. fragrance* sex-linked region, including 5 genes, is micro-syntenic to a region on *M. yunnanensis* chromosome 3 (Fig. 2D), but adjacent regions do not show any synteny. It is possible to hypothesise that this region on chromosome 3 corresponds the sex-determining region in *M. yunnanensis*, but this cannot be confirmed without additional work in that species. The sex of the plants used for sequencing of the *M. yunnanensis* genome is not known because the plants were too young to flower (Jing Cai, personal communication), making it impossible to identify the sex chromosome in this species based on published data.

### Identification of the sex-linked region

To identify the location of the sex-linked region in the nutmeg genome, we used three complimentary approaches based on the analyses of single-nucleotide polymorphisms (SNPs) and sequence read coverage using Illumina genome resequencing data from seven males and eleven females. In total, we generated 383 Gb of raw short-read data (Supplementary Table S5). On average, 45.02 Gb (±5.32, SD) of genome sequence data were obtained per individual. These data were used to read mapping and SNP calling, with each male haplotype used separately as the reference.

**Fig. 3:**
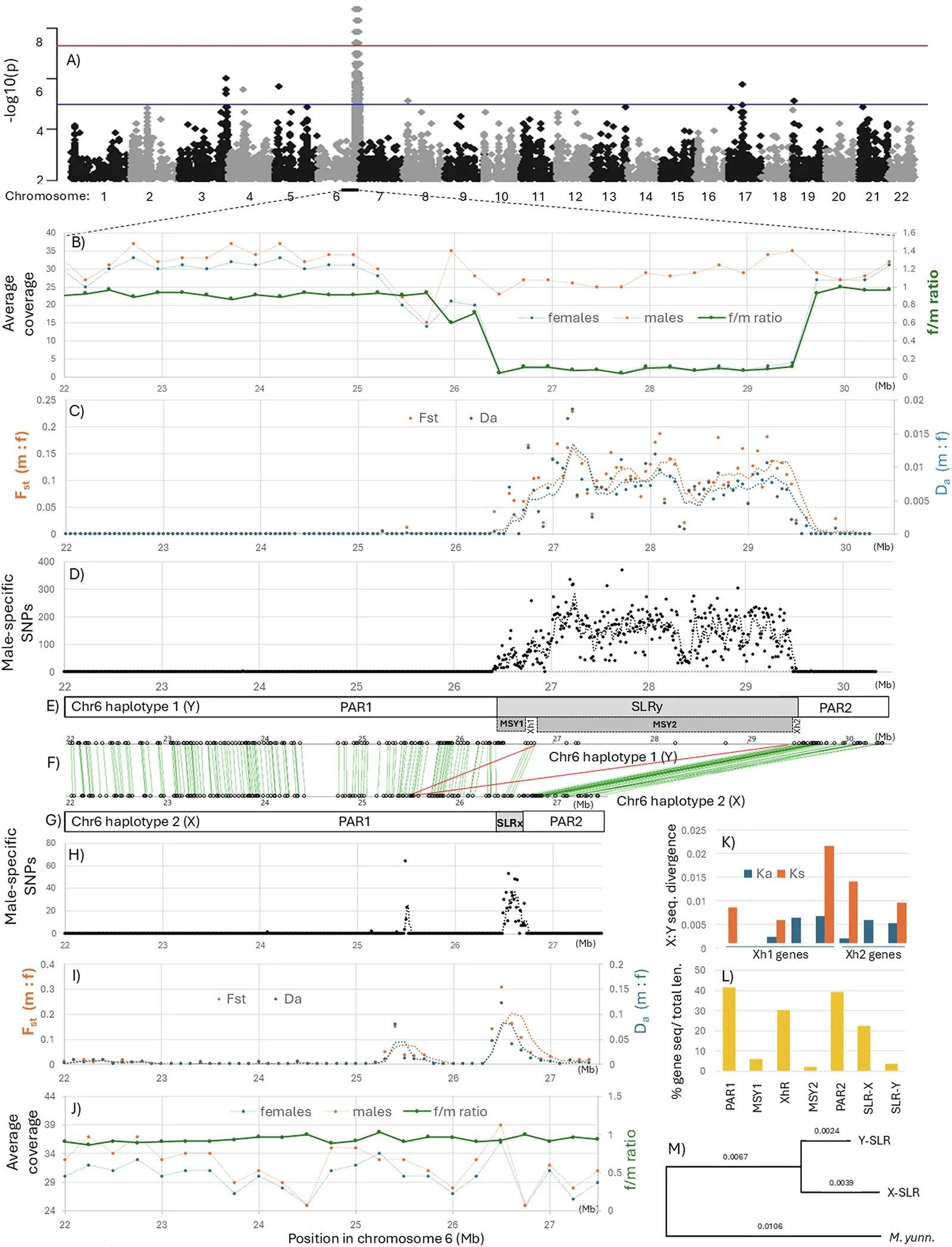
Localisation of the sex-linked region on chromosome 6 of the nutmeg genome. **A**–**E** Use hap1 (Y) as the reference. **G**–**J** use hap2 (X) as the reference. **A** Genome-wide association study using sex as the phenotypic trait. **B**, **L** Short read sequence coverage in 7 males and 11 females using 250 kb long bins. **C**, **K** *F*_st_ between males and females with 100 kb long bins. **D**, **J** *D*_a_ between males and females with 100 kb long bins. **E**, **I** The number of male-specific SNPs per 10 kb bin. **F**, **H** Diagrams of SLRy and SLRx locations. MSY1 and MSY2 represent male-specific Y-linked regions that are absent from the X chromosome, while Xh1 and Xh2 represent Y-linked regions of homology with the X chromosome. **F** Green lines connect homologous genes on the two haplotypes in the proximity of the SLR. Red lines show duplications from SLRy to PAR1 that likely creates spurious signal of sex-linkage at position 25.5 of the hap2. Small black circles on hap1 and hap2 lines show locations of annotated genes. **J** The number of annotated genes per 100 kb and **K** Synonymous and non-synonymous sequence divergence between X- and Y-linked gametologs in Xh1 and Xh2 regions; **L** Percent of sequence length that corresponds to annotated genes in PAR1, MSY1, XhR, MSY2, PAR2, SLR-X and SLR-Y (combined MSY1, XhR and MSY2). **M** NJ phylogeny of nutmeg Y-SLR and X-SLR and outgroup *M. yunnanensis* based on concatenation of CDSs of SLR-Xh1 genes (Fig. 2D) for which Y-, X-linked and outgroup sequences were available.

The GWAS for sex-linkage (with Bonferroni correction, where only SNPs with a p-value lower than 1.49 × 10^−8^ were considered statistically significant) revealed that a ~3.1 Mb region on chromosome 6 of hap1 was statistically significantly associated with sex (Fig. 3A). The 2681 SNPs of interest were clustered on chromosome 6 from 26.47 to 29.57 Mb on hap1. There were no statistically significant SNPs observed in hap2 (Supplementary Fig. S2A).

The average sequencing coverage across all individuals was 36.44 (±7.04, SD) for hap1 and 36.85 (±7.1, SD) for hap2. The coverage analysis revealed a fairly uniform distribution across the genome (Supplementary Fig. S3), with variation mainly in repeat-rich regions, which are known to cause mapping artifacts. However, a ~3 Mb long region on chromosome 6 of hap1 showed a stark difference in coverage between sexes, where all female individuals had a coverage close to 0, while male individuals maintained levels similar to surrounding regions, indicating this region is likely sex-linked and male-specific (Fig. 3B). This pattern was absent in hap2 (Fig. 3J), indicating that *M. fragrans* is male heterogametic, and that chromosome 6 of hap1 contains the Y-SLR (Fig. 3F) whereas the corresponding region of hap2 is the X-SLR (Fig. 3H).

The average *F*_st_ values between males and females at the chromosome and genome level were close to 0, but was elevated around the putative sex-linked regions on chromosome 6 on both hap1 (Fig. 3C) and hap2 (Fig. 3I). Net sequence divergence (*D*_a_) between males and females was near zero all over the genome, except the Y-SLR (Fig. 3C) and X-SLR (Fig. 3I) on chromosome 6.

To further confirm the location of the sex-linked region and to pinpoint its boundaries, we analysed the distribution of sex-specific SNPs. For the SNPs called using hap1 (Y) as the reference, we required all females to be homozygous for the non-reference allele ('ALT' allele in the VCF file), while the males needed to be heterozygous. For hap1, such male-specific SNPs were found only between positions 26.47 and 29.57 Mb of chromosome 6 (Fig. 3D). It is worth noting that while the average sequence coverage in females for this region was low (Fig. 3B), it was not zero, allowing us to call SNPs at some positions in females in this region.

For the SNPs called using hap2 (X) as the reference, we required all females to be homozygous for the reference allele, while all males needed to be heterozygous. Such SNPs were found only between positions 25.4 and 26.7 Mb on the hap2 of chromosome 6 (Fig. 3H). They form a major peak around position 26.5 Mb and a minor peak around positions 25.5 Mb. The minor peak between positions 25,474,965 and 25,506,534 includes 64 male-specific SNPs, while the major peak between positions 26,488,672 and 26,705,386 includes 278 male-specific SNPs. Two peaks with similar positions are also visible in *D*_a_ and *F*_st_ plots (Fig. 3I). Manual checking of male-specific SNPs revealed that the left peak is a false-positive caused by fragmented paralogous pseudogenes homologous to positions 26.47 Mb and 29.57 Mb in Y-SLR (shown with red lines on Fig. 3F).

The results of the analyses reported above indicate that the region between 26.47 and 29.57 Mb of the chromosome 6 hap1 corresponds to the Y-SLR, which is 3.1 Mb long, while the X-SLR is located between positions 26.49 and 26.72 and is only 0.28 Mb long. The difference in length between the X- and Y-linked SLRs is due to the presence of male-specific Y-linked (MSY) regions in the haplotype 1 (Fig. 3E). The rest of the chromosome 6 is likely pseudoautosomal, as evidenced by complete collinearity (Fig. 3F), lack of sequence divergence and sex-specific SNPs in the regions to the left (PAR1) and right (PAR2) of the sex-linked region (Fig. 3E, G).

### Composition of the sex-linked region

Eight and 32 intact genes were annotated within the SLR on the X and Y haplotypes, respectively. The identified sex-linked genes had various functions, including transcription, cellular processes, respiratory processes, biosynthetic processes and developmental processes (Supplementary Table S6). Several genes were identified as having protein domains related to plant development, seed and flower growth and hormonal regulation (KH, Zf-CCHC and DnaJ protein domains), of which only the DnaJ protein domain gene was found in the X- and Y-SLR. However, a more detailed functional analysis of these genes would be necessary to further explore their function and study their possible role in sex determination.

The comparison of gene order along the two haplotypes of chromosome 6 revealed nearly complete collinearity, except the Y-SLR, where 24 Y-linked genes lacked gametologs in hap2 (Fig. 3F). The male-specific genes in Y-SLR are arranged in two blocks that we called MSY1 and MSY2 (Fig. 3E). MSY1 and MSY2 are separated by a 101-kb long X-homologous region 1 (Xh1) that contains 6 genes. Five of these genes have gametologs in the X-SLR (Fig. 3F) and homologues in the outgroup species *M. yunnanensis* (Fig. 2D). Phylogeny for these genes, rooted by the outgroup sequences, shows that X- and Y-linked genes of *M. fragrans* cluster together, while the outgroup sequence is more divergent and equidistant from the X- and Y-linked genes (Fig. 3M). The clustering of the *M. fragrans* X- and Y-linked genes implies evolution of sex linkage in this region after divergence from *M. yunnanensis*, but other parts of the *M. fragrance* SLR may be considerably older.

The second region of homology with the X-SLR (Xh2) is present at the end of Y-SLR (Fig. 3E). This region contains three genes, all of which have intact X- and Y-linked gametologs. Nucleotide divergence between X- and Y-linked gametologs in both Xh1 and Xh2 regions is low, *K*_s_ < 2.5% for most X:Y pairs (Fig. 3K), indicating recent cessation of X:Y recombination, or a translocation from one to the other sex chromosome. Given that the collinearity of hap1 and hap2 is preserved in this region, the translocation is a less likely explanation than the recent cessation of recombination due to the expansion of the non-recombining sex-linked region.

**Fig. 4:**
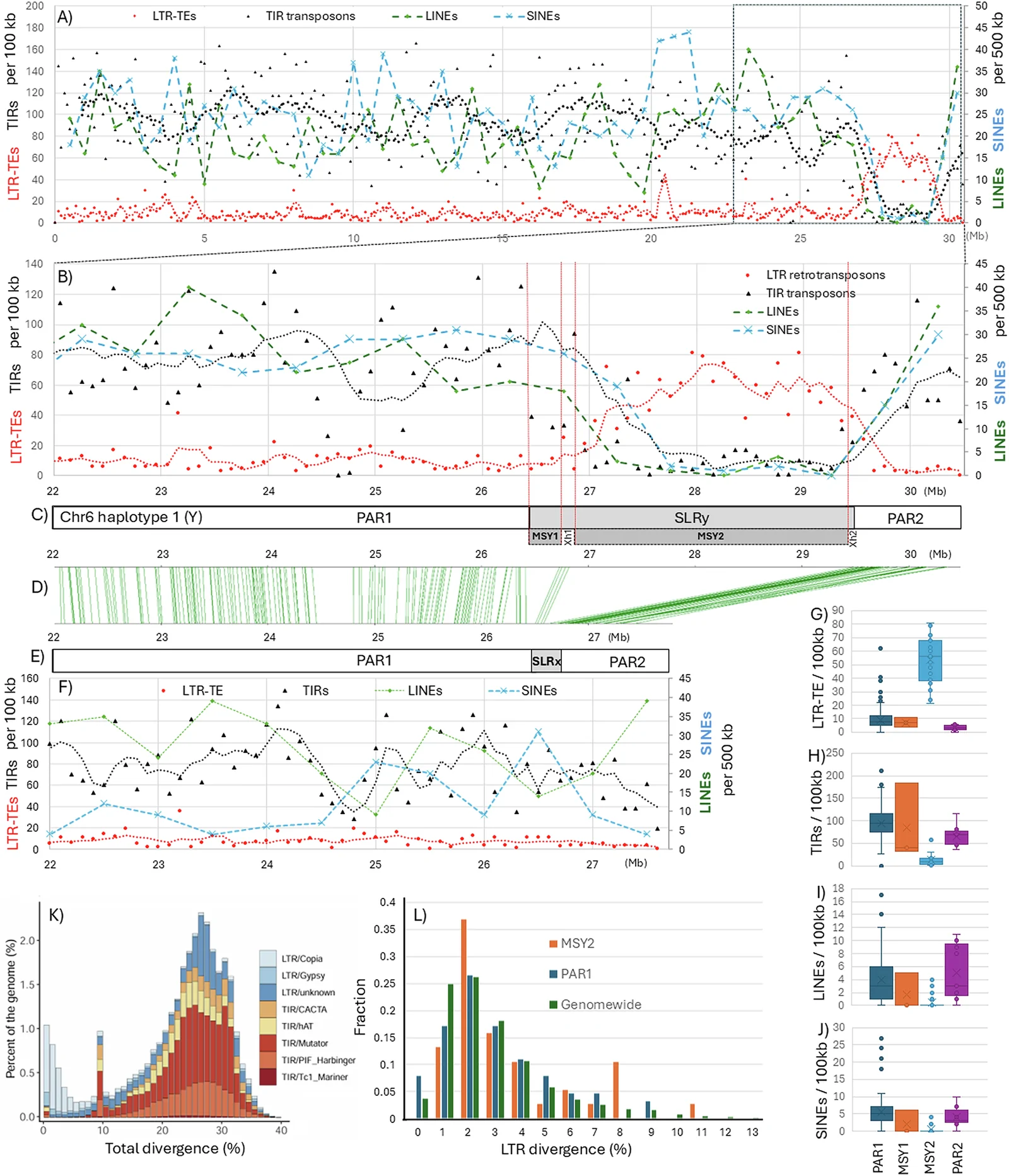
Distribution of TEs in and around SLR on nutmeg chromosome 6. **A**–**C**, **G**–**J** Represent hap1 (Y), while panels E and F represent hap2 (X). **A** Shows the entire length of hap1, while **B** shows only the region distal to position 22 Mb of chr6 with higher resolution. In **A**, **B**, **F** the left axis shows the number of LTR retrotransposons or TIR transposons per 100 kb, while the right axis shows the number of LINEs and SINEs per 500 kb. The dotted, dashed and solid lines on **A**, **B**, **F** represent 5 data point moving average, except the LINEs and SINEs in (**A**, **B**), where the lines connect the adjacent data points. **C**, **E** Show diagrams of SLRy and SLRx locations. MSY1 and MSY2 represent male-specific Y-linked regions that are absent from the X chromosome, while Xh1 and Xh2 represent Y-linked regions of homology with the X chromosome. **D** Green lines connect homologous genes on the two haplotypes in the proximity of the SLR. **G**–**J** Compare the density of different types of TEs per 100 kb between the PAR1, MSY1, MSY2 and PAR2. MSY2 is significantly (chi^2^, *P* < 0.01) different from other regions on all four plots. The error bars above and below the boxes show the range of values after outlier exclusion, while the box shows the first and third quartiles as well as the median, as standard in boxplots in R. **K** Percent of the genome represented with different types of TEs plotted against total sequence divergence between the copies of each element type. Note the predominance of LTR retrotransposons with low sequence divergence at the left of the plot. **L** The distribution of sequence divergence between LTRs of the same copy of the element (proxy for age of insertion) in the MSY2, PAR1 and genome-wide. Note that MSY1, XhR and PAR2 are not shown due to too few TE copies with both LTRs present.

The analysis of TEs revealed that MSY2 is significantly enriched with LTR retrotransposons, while LINEs and TIRs are significantly depleted in this region compared to the adjacent regions (Fig. 4) and the rest of the genome. TEs often accumulate in non-recombining regions, but TE density in MSY1 is similar to that in the PAR1 and significantly differs from MSY2 (Fig. 4), suggesting that MSY1 is a recent addition to the SLR, while MSY2 has lacked recombination for a longer period of time. This is consistent with low nucleotide divergence (*K*_s_) between X- and Y-linked gametologs of genes in the region between the MSY1 and MSY2 (Fig. 3K). We hypothesise that the MSY2 represents the older stratum, while MSY1 and XhR evolved sex linkage in the last ~5 million years (=*g K*_s_/2 *m*), based on average X:Y *K*_s_ = 0.7% for sex-linked genes with homologues present on the X and the Y chromosomes (Fig. 3K) and assuming *g* = 10 years per generation (as nutmeg first flowers at the age ~8 years) and *m* = 7 × 10^−9^ per nucleotide per generation mutation rate (see Table 1 in Krasovec et al.^55^).

The distribution of sequence divergence between copies of TE of the same family across the genome reveals that most TE families were active a long time ago, with the major peak of divergence at ~28% (Fig. 4K). There is also a distinct peak including multiple types of TEs at ~10% sequence divergence, indicating a short burst of TE activity around 71 million years ago (MYA), assuming 10 years generation time and 7 × 10^−9^ per nucleotide per generation mutation rate, based on the mutation rates reported for various angiosperms (see Table 1 in Krasovec et al.^55^). However, it is worth noting that generation time has likely changed over evolutionary time, and the mutation rate for TE may differ from nuclear genes used by Krasovec et al.^55^, creating considerable uncertainly in dating ancient bursts or TE activity. LTR retrotransposons, dominated by copia, show a peak of recent activity (left peak at Fig. 4K), indicating that currently, or very recently, this TE is the most actively transposing type in the nutmeg genome. The divergence between two LTRs of the same copy provides a proxy for time since insertion of individual retrotransposon copies. The distribution of LTR divergence peaks at 2% (Fig. 4L), indicating that these TEs were most active around 14 MYA, and since then its activity declined. Interestingly, the MSY2 peak at 2% LTR divergence is higher than the rest of the genome, indicating preferential accumulation of LTR retrotransposons in the non-recombining region. Assuming that the accumulation of LTR-TE in this region was caused by recombination cessation, it is possible to infer that MSY2 is at least 14 MY old. The other types of TEs are underrepresented in the MSY2 (Fig. 4B), possibly because they were inactive in the time since this region evolved recombination cessation and accumulation of LTR retrotransposons, ‘diluted’ their abundance. This interplay of TE activity/inactivity and the evolution of recombination cessation in the Y-SLR can also explain no excess of LTR retrotransposons in the MSY1 and XhR if these regions evolved sex linkage more recently than the time when LTRs were actively transposing.

### Y-chromosome degeneration

Y-SLR contains 32 annotated protein-coding genes, 8 of which have annotated homologues in X-SLR. Synonymous site divergence (*K*_s_) for the pairs of homologous X- and Y-linked genes is low (Fig. 3K), suggesting recent cessation of recombination between X- and Y-linked gametologs due to inclusion of these genes in the sex-linked region. Y-degeneration can lead to complete or partial gene loss from the Y-chromosome. However, all 8 X-SLR genes have intact Y-linked homologues. Gene loss and accumulation of repetitive DNA typically lead to lower gene density on the Y-chromosome compared to the X and non-sex-linked regions. Consistent with this, gene density in X-SLR is not significantly different from the genome-wide average of ~31 genes/Mb, while gene density of the Y-SLR is much lower (Fig. 3L), likely due to gene loss and accumulation of TEs.

Sequence analysis identified 91 pseudogenes in the Y-SLR, totalling 34,746 nucleotides in length. Half (46 out of 91) of the pseudogenes had homology to Y-SLR genes with a mean sequence identity of 91.6%. These pseudogenes represent partial duplicates of Y-SLR genes. The other half were homologues of annotated genes elsewhere in the genome, with a mean sequence identity of 77.7%. No Y-SLR pseudogenes homologous to X-SLR genes were detected. The pseudogene density in the Y-SLR (30.3 pseudogenes per Mb, with total length 12 kb) was three times higher than observed outside Y-SLR on the same chromosome (10.5 pseudogenes per Mb, with total length 3.5 kb). Most pseudogenes (78) were located in MSY2 (31.2 pseudogenes per Mb), while only 2 pseudogenes were found in MSY1 (7.6 pseudogenes per Mb). This pattern is consistent with a longer period of recombination suppression and accumulation of pseudogenes in MSY2, suggesting that MSY2 represents an older sex-linked region.

**Fig. 5:**
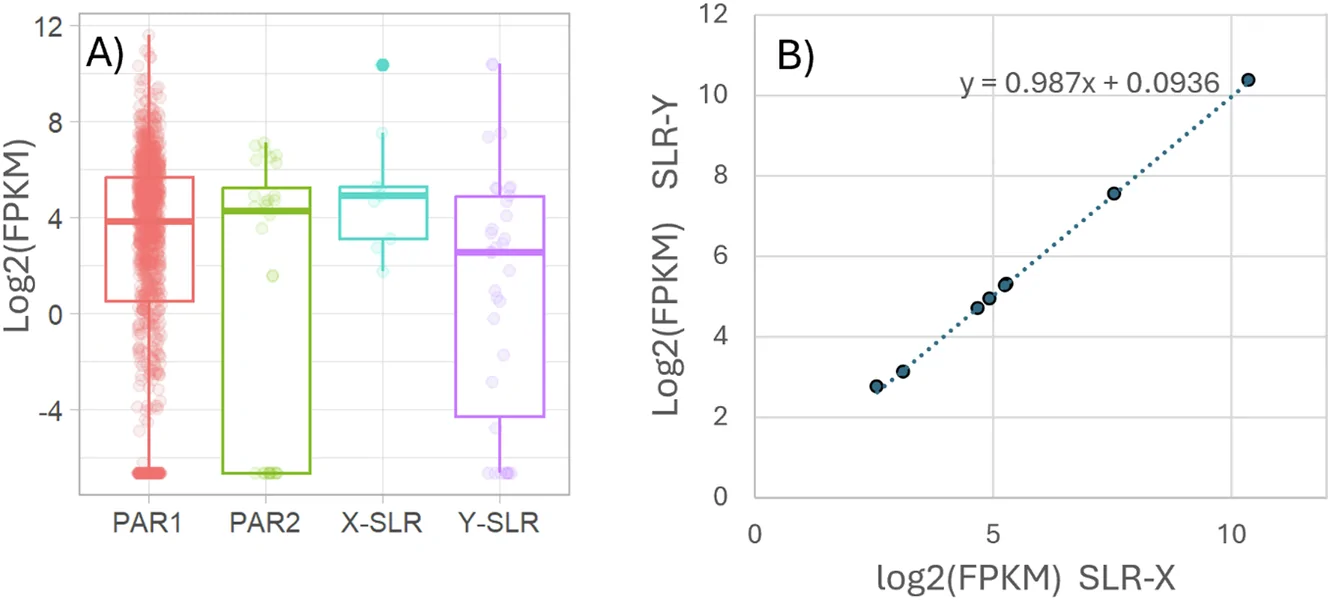
Expression of sex-linked and pseudoautosomal genes. **A** The comparison of gene expression (log2(FPKM)) in the pseudoautosomal regions and the X- and Y-linked genes. The difference between these groups of genes is non-significant. The boxplot shows the actual data points as circles, the error bars above and below the boxes show the range of values after outlier exclusion, while the box shows the interquartile range and median. **B** The comparison of gene expression between Y-linked genes and their X-linked gametologs. Gene expression is almost identical in the Y-linked genes and their X-linked gametologs.

The comparison of synonymous / non-synonymous rate ratios (*K*_a_/*K*_s_) did not reveal a significant difference between the X- and Y-linked genes for which orthologs from outgroup *M. yunnanensis* were available. The analysis of gene expression revealed that median expression of Y-linked genes is somewhat lower compared to X-linked and pseudoautosomal genes (Fig. 5A), but this difference is not significant. No expression was detected in 8 out of 32 annotated Y-linked genes, while all X-linked genes were expressed. The proportion of unexpressed genes in MSY is only marginally (*P* = 0.076, 2 × 2 chi^2^ = 3.1) significantly higher compared to the proportion of unexpressed pseudoautosomal genes (183 out of 1129). The Y-linked genes lacking expression are located in MSY1 and MSY2 (3 and 5 genes, respectively), while for the 8 genes with X- and Y-linked gametologs, expression was almost identical (Fig. 5B), revealing no evidence for Y-degeneration in gene expression. Thus, the only evidence for Y-degeneration we found in nutmeg is the accumulation of TE and pseudogenes, which likely contributed to the increase of Y-SLR size.

### Development of a sex-specific PCR marker for sexing nutmeg seedlings

To provide a reliable marker to sex nutmeg seedlings in agriculture, we developed a sex-specific PCR marker. The availability of molecular markers to reliably distinguish male from female seedlings is important in nutmeg production, as it can reduce costs related to growing unproductive individuals (i.e. males). To develop a sex-linked marker, we tested 26 primer pairs (Supplementary Fig. S5 and Table S7 for primer sequences and S8 for PCR protocol) and found one pair that reliably distinguished males from females (Supplementary Fig. S5). Primer pair Mf_7216M2 amplifies ~0.6 kb long fragment of the Y-SLR gene g7232 (located at position 29.489 Mb in hap1) in males and not in females. It is worth mentioning that this marker also amplifies in monoecious individuals (marked 'H' in Supplementary Fig. S5) that occasionally appear in nutmegs. This observation suggests that monoecious nutmeg plants may carry Y-linked sequences and could represent either unstable males or individuals with a modified Y chromosome that has reduced ability to suppress the development of female reproductive organs, similar to the Yh chromosome in papaya^56^.

## Discussion

In this study, we present the first assembly and analysis of *Myristica fragrans* 0.6 Gb long genome, which is on the lower side of the range of genome sizes in spermatophytes and is within the genome size range of other magnoliid species^57^. The GC-content, gene and TE density in the nutmeg genome is more evenly distributed compared to most other sequenced plant genomes that typically have low gene density in a fairly large central (usually pericentromeric) part of the chromosomes. The even distribution in nutmeg likely reflects the lack of a single localised centromere and pericentromeric region in the holocentric chromosomes. Holocentric chromosomes, characterised by a diffuse centromere, are uncommon in eukaryotes^36^, and the impact of holocentricity on the evolution of sex chromosomes is not well understood.

The newly sequenced nutmeg genome was used to locate and characterise the SLR in *M. fragrans*. Our finding that this species has a male-heterogametic (XY) reproductive system contradicts the conclusion of the previous RAPD-based study that reported the presence of female heterogamety in *M. fragrans*^34^. However, RAPD markers can be unreliable, while our conclusion is based on several complimentary approaches (*F*_st_, *D*_a_, presence of male-specific SNPs, GWAS and sequence coverage) that all consistently support male heterogamety and the location of SLR close to one end of chromosome 6. The Y-SLR is ~3 Mb long, located between positions 26.47–29.57 Mb on haplotype 1 of chromosome 6. The X-SLR is much shorter —only 0.23 Mb and located between positions 26.49 and 26.72 Mb on haplotype 2 of chromosome 6. This difference in size between the Y- and X-linked SLR is due to the presence of a Y-specific region. It seems plausible that deletion of this region from the nascent X chromosome may have played a key role in the evolution of sex chromosomes in nutmeg, though we cannot rule out an alternative scenario that the absence of this region is ancestral and it evolved on the Y chromosome. As dioecy is ancestral in this plant family, there are no sufficiently close outgroup species to provide information about the likely ancestral state and distinguish these scenarios of MSY evolution.

The genome analysis of the closely related dioecious *M. yunnanensis* revealed considerable synteny preservation across many chromosomes, but no macrosynteny was found for the nutmeg sex chromosome (chr6) that has small homologous regions scattered across many *M. yunnanensis* chromosomes (Fig. 2C). The nutmeg Y-specific region was not found in *M. yunnanensis*, suggesting that the sequenced genome belongs to a female (sex of the sequenced individual was not reported in ref. ^54^). Interestingly, the homologues of nutmeg sex-linked genes in *M. yunnanensis* are equidistant from X- and Y-linked gametologs (Fig. 3M) suggesting that at least the genes in Xh1 region, and possibly the entire nutmeg SLR evolved sex-linkage after the divergence between *M. fragrans* and *M. yunnanensis*. If the nutmeg SLR evolved since the divergence from *M. yunnanensis*, then there must have been a recent sex chromosome turnover, and a sex-linked region in *M. yunnanensis* would be expected to be located in a different region of the genome. Lack of synteny for the nutmeg chromosome 6 with *M. yunnanensis* chromosomes is consistent with recent sex chromosome turnover. Such turnovers are common in dioecious plants^3,13^.

However, the high density of repeat elements, pseudogenes and the lower density of genes than the genomic average on the Y chromosome (Fig. 4) suggest that the SLR on chromosome 6 is at least a few million years old. X:Y divergence for the SLR genes with shared X- and Y-linked homologues is quite low (*K*_s_ < 0.025, Fig. 3K), suggesting that nutmeg sex chromosomes are of recent origin. However, these genes may be located in the region recently added to the sex-linked region (i.e. it is a recent stratum), while the MSY2 may be older, as suggested by the prevalence of dioecy in the entire family Myristicaceae. The absence of X-linked homologues for the Y-linked genes in the MSY region does not allow us to estimate the age of nutmeg sex chromosomes more accurately. Identification of SLR in more Myristicaceae species will reveal how long ago the sex chromosomes have evolved and whether they have undergone any turnovers in this family.

Although other groups of land plants, such as bryophytes, have ancient sex chromosomes^58^, most dioecious angiosperm species have SLRs of a fairly recent origin (e.g. *Amborella trichopoda*^59^), wherein the SLR is relatively small with little to no accumulation of repetitive sequences. However, there are a few exceptions, such as *Silene latifolia*, where the X and Y sex chromosomes are heteromorphic, and the oldest sex-linked region is estimated to be 11 Ma^26,55^. We could not definitively identify the sex-determining genes in *M. fragrans*. However, the two F-box genes in SLR may have a potential role in floral development, a feature that is crucial in dioecy. To our knowledge, this is the first time that this group of proteins has been found in the SLR, although four MADS-box genes have been identified in *Ginkgo biloba*^60^. MADS-box proteins are another large domain of proteins that has a role in floral development. Most of the other genes annotated in the SLR have an unknown function (Supplementary Table S7).

We identified 10 and 13 male-specific genes in *M. fragrans* MSY1 and MSY2 regions, respectively. While being male-specific, most of these genes have divergent non-sex-linked paralogs elsewhere in the genome, which created difficulties in developing male-specific primers. The Y-linked gene g7232 that was used for primer development is located at the border between the MSY2 and Xh2, and it has an incomplete broken copy in the X-SLR that is sufficiently divergent from g7232 to allow us to design male-specific primers, which will likely be useful for nutmeg growers to sex the seedlings in order to optimise the sex ratio in nutmeg plantations. It is worth mentioning that the previously published supposedly sex-specific markers from Shibu et al.^34^ were not sex specific in our samples (Supplementary Fig. S6).

To our knowledge, this is the first species within the whole Myristicaceae family for which the sex chromosomes have been identified and characterised. Considering that the predominant reproductive system within the family is dioecy, it could be presumed that this is the ancestral state of the family, with monoecy being the derived state. However, taxonomic descriptions of several species are inconsistent with field observations, calling for a revaluation of the reproductive systems within the family. For example, *M. insipida, M. fragrans and Iryanthera hostmannii* are considered dioecious, but have reports of monoecious individuals^61–63^. In *Myristica fragrans*, occasional reports of individuals bearing both male and female flowers are more plausibly explained as instances of *leaky dioecy* rather than evidence for stable monoecy. Similar leakiness has been increasingly documented in other dioecious angiosperms with sex chromosomes^64^, including the well-studied case of *Mercurialis annua*, where environmental factors such as pollen limitation can induce opposite-sex flower production^65^. In nutmeg, such plasticity may function as a reproductive assurance mechanism under conditions of mate or pollen scarcity, while the species retains an underlying dioecious sexual system.

### Concluding remarks

We produced the first chromosome-level haplotype-resolved assembly of a male nutmeg (*M. fragrans*) and identified chromosome 6 as the sex chromosome, with a 3 Mb long sex-linked region. While the age of the nutmeg SLR cannot be established, it is clear that at least part of this region, including the X-homologous region, evolved sex-linkage recently (since nutmeg divergence from closely related *M. yunnanensis*). The rest of SLR is probably older and may have evolved as a result of deletion(s) on the nascent X chromosome, making most of SLR male-specific, which should prevent recombination on the Y-chromosome. It is tempting to speculate that deletion(s) of this region on the X chromosome may have been the cause of initial recombination suppression between the proto-X and proto-Y chromosomes. Identification and characterisation of SLR in other nutmeg species will help to shed light on the origin and evolutionary dynamics of sex chromosomes in this primarily dioecious, understudied plant family.

## Methods

### Genome size estimation

Genome size was estimated with flow cytometry using nutmeg young leaves, with tomato (*Solanum lycopersicum*, genome size ≈ 900 Mb) and maize, inbred line B73 (*Zea mays*, genome size ≈ 2.3 Gb) as internal standards. Fresh tissue was chopped in ice-cold Mg²⁺ buffer, filtered through a 40-μm nylon mesh, and nuclei were stained with propidium iodide (50 μg/mL) in the presence of RNase A. Stained nuclei were analyzed on a BD FACScalibur flow cytometer with 488-nm excitation, collecting at least 10,000 nuclei per sample, and data were processed with ModFit LT v3.0. Genome size was calculated from the ratio of mean fluorescence intensities of the sample and the internal standard, multiplied by the known genome size of the standard.

### DNA extraction and sequencing for reference genome assembly

The male individual used to produce the reference genome in this study was grown at the Xinglong Tropical Botanical Garden (Wanning, Hainan Province, PR of China). High molecular weight DNA was isolated from fresh young leaves of a single male plant using the CTAB method: the sample was ground in liquid nitrogen, incubated with CTAB buffer, and extracted with chloroform/isoamyl alcohol (24:1). The DNA was then precipitated with isopropanol and sodium acetate, washed with 75% ethanol, air‑dried, dissolved in TE buffer, and stored at −20 °C. Genomic DNA quality was assessed using 1% agarose electrophoresis. Genomic DNA was sheared using g-Tubes (Covaris) and concentrated with AMPure PB magnetic beads. Then, the DNA was quantified using the Qubit TM3 Fluorometer (Invitrogen). The SMRT bell library was constructed using the SMRTbell® Express Template Prep Kit 2.0, followed by primer annealing and binding of SMRT bell templates to polymerases with the DNA/Polymerase Binding Kit. Sequencing was performed on the Pacific Biosciences Sequel II platform (Annoroad Gene Technology) to generate HiFi reads. For Hi-C library construction, young leaves were cross-linked with 2% formaldehyde at ambient temperature in a hybrid rotary hearth furnace for 90 min. Cells were lysed and digested using the Hind III restriction enzyme. DNA ends were filled and labelled with biotin. Hi-C libraries were created using the Mate-pair Kit. Complexes containing the biotin-labelled ligation products were purified and sheared to 300–700 bp, followed by pulling down. Paired-end reads (2 × 150 bp) were sequenced on an Illumina HiSeq X Ten platform, yielding a total of 66,928,937,824 bp (Supplementary Table S1). The resulting HiFi and Hi-C data are available from the European Nucleotide Archive under project number PRJEB112374.

### Genome assembly and annotation

We produced a haplotype-resolved male genome assembly using Hifiasm^66^ (version 0.16.1, with parameter -*s* = 0.2) in combination with Hi-C reads, while the Hi-C reads were assembled with HiC-Pro^67^ (version 3.1.0, with default parameters). Duplicate reads were removed with Khaper^68^ (the program developer did not provide version information), and reads were polished with NextPolish2^69^ (version 0.2.1, with default parameters). Finally, EndHiC^70^ (version 1.0.0, with parameter --rounds = 3, --binnumstep = 5) was used to anchor the chromosomes.

Transposable elements were identified and quantified using the default parameters of EDTA v2^71^ with the parameter *--cds*, which allows the pipeline to exclude coding regions from the TE library. Structural gene prediction and annotation was performed using BRAKER3 v3.0.8^46^ by combining the RNA sequences, the soft-masked genome produced by EDTA, and the Viridiplantae protein dataset. Functional annotation was performed using emapper v2^72^ and OrthoDB^73^. Pseudogenes were identified using PseudogenePipeline^74^.

### Genome resequencing

Short-read genome sequencing was done for 18 individuals of known sex: seven males and eleven females. Samples for 16 individuals were kindly provided by the Singapore Botanic Garden (Republic of Singapore), and two individuals were obtained from the Eden Project (Cornwall, United Kingdom) (Supplementary Table S5). Total DNA was extracted from silica-dried leaves following the MATAB protocol as described in ref. ^75^. DNA was quantified using Qubit 2.0 (Invitrogen). Genomic libraries were constructed at Azenta Life Sciences (Germany), and short-read (150 bp) sequencing was performed on an Illumina NovaSeq platform at the same facility with at least 30x coverage. The sequence data for 11 females and 7 males are available from NCBI SRA under project number PRJNA1301071.

### RNA extraction and sequencing

Total RNA was isolated from fresh leaves from the same male individual that was used for sequencing the reference genome, using the TRIzol reagent (Thermo Fisher Scientific) following the manufacturer’s protocol. To account for any variability in environmental conditions, we sampled and sequenced three replicates per tissue, with sampling done at different days. The concentration of extracted nucleic acids was detected with the Nanodrop2000 Spectrophotometer (Thermo Fisher Scientific). RNA concentration and integrity were quantified with the Agilent 2100 RNA nano 6000 assay kit (Agilent Technologies). RNA-seq library construction was performed from extracted samples, and libraries were sequenced on the Illumina NovaSeq platform with the NovaSeq 6000 S4 Reagent Kit. The analysis of gene expression was conducted with RSEM^76^, using gene sequences from the annotated genome as the reference for RNA-seq read mapping.

### Bioinformatic processing

Raw reads were trimmed and filtered with fastp v0.23.4^77^, and then mapped to haplotypes 1 (hereafter hap1) and 2 (hereafter hap2) of the male reference using bowtie2 v2.5.4^78^. Mapped reads were then sorted, indexed and piled up using the *sort*, *index* and *mpileup* commands of samtools v1.7^79^. Variant calling was performed with vcftools v0.1.16^80^, and initial filtering was done with bcftools v1.21^81^. After the quality of the data was visually inspected, vcftools was used to filter out indels, multiallelic sites, sites with more than 80% missing data, sites with coverage less than 5, and a minor allele frequency of at least 0.1.

### Identification of a candidate sex-linked region

As there is no prior knowledge on the size and age of the SDR, we used several approaches for SDR identification. First, we explored the datasets by performing a coverage analysis on the mapped reads using mosdepth v0.3.8^82^ with 250 kb sliding windows. All polymorphism analyses, including calculation of *D*_a_ and *F*_st_ between males and females, were done in proSeq4^83^. In addition, we performed a genome-wide association study (GWAS) with plink v1.9^84^, where we defined sex as the phenotypic trait and used the *-assoc* parameter for a basic association study. Finally, we identified sex-specific SNPs (i.e. with one of the alleles present in one but not the other sex) using filtering of SNPs in Microsoft Excel.

### The comparison with the congeneric genome

Synteny between *M. fragrans* and *M. yunnanensis* genomes was analysed with MCScanX^85^ and two-way nucleotide blast^86^ for annotated coding regions. The sequences for coding regions from 1-to-1 orthologs of the two species were aligned in ProSeq4^83^. The same program was used to check the alignments and calculate pairwise synonymous (*K*_s_) and nonsynonymous (*K*_a_) divergence between the species.

### Phylogenetic analyses

Phylogenetic analysis for concatenated coding regions of genes with available X-, Y-linked and outgroup *M. yunnanensis* orthologs was conducted in MEGA^87^. Synonymous site divergence was estimated using the Nei-Gojobori method with Jukes-Cantor distances for synonymous sites. The phylogeny-based comparison of substitution rates in the X- and Y-linked genes with outgroup sequence available were conducted in the codeml program from the PAML package^88^. Omega (*K*_a_/*K*_s_) ratio was estimated in the branch model, allowing for separate rates for X- and Y-linked genes. For testing the significance of *K*_a_/*K*_s_ ratios between the branches, we used a likelihood ratio test comparing log-likelihoods for two branch models, one that allows different rates for X- and Y-linked genes and the model allowing these branches to have the same rate.

### Development of sex-specific PCR markers

We developed sex-specific primers to validate the identified SLR. More specifically, we checked which genes within the SLR were present in one sex, but not the other. Then, we chose one gene of interest and used blast^86^ to query the gene sequences against the genome to verify that the sequence was specific to only that region. If the query had a high similarity to another region in the genome, we developed primers so that at least one of the last five bases on the 3′ end is different in the other region. Theoretically, this should ensure a high specificity of the primers. Additionally, we tested previously developed sex-specific primers for *Myristica fragrans*^34^. We first tested each developed primer on two males and two females. The primers that were highly sex-specific were then used on all available samples. The PCR conditions are listed in Supplementary Table S8. Each 50 µL PCR reaction contained approximately 14–15 ng of genomic DNA and 29–40 ng of each primer (equivalent to 2.5 pmol per primer, depending on primer length).

### Reporting summary

Further information on research design is available in the Nature Portfolio Reporting Summary linked to this article.

## Supplementary information

Supplementary Information

Reporting Summary

## Data Availability

Genome and transcriptome sequence data generated in this project are available from NCBI under project number PRJNA1301071. The genome assembly data are available from the European Nucleotide Archive (https://www.ebi.ac.uk/ena/browser/home) under project number PRJEB112374. The genome assemblies are also available in the NGDC Genome Warehouse repository (https://ngdc.cncb.ac.cn/gwh/) under project number PRJCA063189.
