# Supplementary material for: A haplotype-resolved nutmeg genome reveals XY sex chromosome evolution and enables molecular sexing
Source: Commun Biol. 2026 May 28;9:1132. doi: 10.1038/s42003-026-10355-0 (PMC13500479; doi:10.1038/s42003-026-10355-0)
Supplement: Supplementary file 1 [file 42003_2026_10355_MOESM1_ESM.pdf]

Supplementary Information

A haplotype-resolved nutmeg genome reveals XY sex chromosome evolution and enables molecular sexing

Katarina Matvijev[1,#], Sufang Wang[1,2,#], Mengjun Xiao[3,#], Ashini D. Mahadura[1,4], Ray Ming[3**] and Dmitry A. Filatov[1*]

[1] Department of Biology, University of Oxford, South Parks Rd, Oxford OX1 3EL, United Kingdom

[2] School of Life Science and Technology, Northwestern Polytechnical University, 1 Dongxiang Road, Xi'an, Shaanxi, 710129, China

[3] Centre for Genomics and Biotechnology, Fujian Provincial Key Laboratory of Haixia Applied Plant Systems Biology, Key Laboratory of Genetics, Breeding and Multiple Utilization of Crops, Ministry of Education, Fujian Agriculture and Forestry University, Fuzhou, 350002, China

[4] Department of Botany and Zoology, Faculty of Science, Masaryk University, Brno, Czech Republic.

[#] 1st co-authorship

Correspondence:

[*] dmitry.filatov@biology.ox.ac.uk (ORCID: 0000-0001-8077-5452)

[**] rayming@illinois.edu (ORCID: 0000-0002-9417-5789)

Table S1. Summary statistics from HiFi and HiC sequencing on male sample.

	**HiC**	**HiFi**
Sample name	Unknown_BP185-006H0002	PB_BMK230915-BP185-001P0003
Read pairs	223,495,564	1,282,650
Bases	66,928,937,824	19,542,512,782
%≥Q30	92.96	-
ccs Reads N50 (bp)	-	15,411
ccs Mean Length (bp)	-	15,236
ccs Longest Read (bp)	-	67,223

Table S2. Summary statistics for RNA sequencing data.

BMK ID	Reads	Bases (bp)	Q20(%)	Q30(%)	GC(%)
xz-flower-1	20,528,391	6,137,094,939	97.73	93.89	45.45
xz-flower-2	25,360,520	7,586,385,834	97.88	94.17	45.42
xz-flower-3	21,973,579	6,574,069,037	97.82	94.02	45.49
xz-stem-1	20,173,658	6,036,060,656	97.70	93.70	44.86
xz-stem-2	20,580,715	6,159,187,896	97.95	94.29	44.90
xz-stem-3	22,465,787	6,730,546,848	97.60	93.39	44.76
xz-leaf-1	20,260,575	6,070,314,431	97.87	94.00	44.42
xz-leaf-2	20,069,332	6,010,869,555	97.63	93.49	44.50
xz-leaf-3	19,664,142	5,883,378,932	97.65	93.53	44.61

Table S3. Chromosome lengths of hap1 and hap2.

Chromosome	**Hap1**	**Hap2**
Chr01	44,078,094	43,169,371
Chr02	35,916,195	33,860,491
Chr03	34,473,871	35,898,566
Chr04	30,689,418	33,537,517
Chr05	31,941,831	32,159,418
Chr06	30,402,304	27,520,841
Chr07	30,819,958	30,494,363
Chr08	30,540,083	30,912,998
Chr09	28,729,926	28,361,111
Chr10	27,088,625	26,486,584
Chr11	26,523,481	26,460,922
Chr12	24,590,506	26,086,129
Chr13	23,850,291	19,154,713
Chr14	24,308,111	24,549,575
Chr15	23,090,109	21,840,786
Chr16	24,114,653	24,761,858
Chr17	24,176,146	23,808,127
Chr18	28,093,337	29,790,251
Chr19	23,242,512	23,915,519
Chr20	23,887,745	16,803,253
Chr21	21,671,624	21,999,239
Chr22	19,027,812	19,098,439
Chr1-Chr22	611,256,632	600,670,071
Unanchored	21,145,503	26,806,527
Total	633,402,135	627,476,598

Table S4. TE annotation summary from EDTA pipeline.

Class	Count	bp Masked	% masked
**Haplotype 1**			
LINE			0.00%
I	43	20472	0.00%
L1	4257	1493209	0.23%
RTE	21321	10123168	1.59%
LTR			
Copia	29097	36599103	5.75%
Gypsy	7828	7947764	1.25%
Unknown	83295	46931824	7.38%
SINE			
Unknown	26049	6007022	0.94%
TIR			
CACTA	53516	12029985	1.89%
Mutator	188447	60243208	9.47%
PIF Harbinger	106085	24889870	3.91%
Tc1_Mariner	6214	1063580	0.17%
hAT	68587	17170216	2.70%
nonTIR			
Helitron	54320	13434767	2.11%
**Total hap1**	**649059**	**237954188**	**37.42%**
**Haplotype 2**			
LINE			
L1	4679	1707552	0.27%
RTE	19817	14420735	2.29%
LTR			
Copia	33018	32647170	5.20%
Gypsy	18064	10194163	1.62%
Unknown	64609	45819679	7.29%
SINE			
Unknown	9365	2744412	0.44%
TIR			
CACTA	62097	15430419	2.46%
Mutator	162306	54409610	8.66%
PIF Harbinger	112661	27914949	4.44%
Tc1_Mariner	2908	683568	0.11%
hAT	86062	21951772	3.49%
nonTIR			
Helitron	63549	14660645	2.33%
**Total hap2**	**639135**	**242584674**	**38.60%**

Table S5. Individuals sampled for genome sequencing and the resequencing summary statistics.

Sample name	Herbarium number	Origin	Sex	Total number of reads	Total number of bases (Gb)	NCBI accession
DF2200	20111530*A	SBG[1)] Singapore	m	300,560,358	45.08	SAMN50434501
DF2201	20111530*B	SBG, Singapore	f	249,066,774	37.36	SAMN50434490
DF2202	20111530*C	SBG, Singapore	f	316,349,532	47.45	SAMN50434491
DF2204	20170578*B	SBG, Singapore	f	301,957,334	45.29	SAMN50434492
DF2206	20180920*B	SBG, Singapore	f	266,102,328	39.92	SAMN50434493
DF2208	20130983*A	SBG, Singapore	m	273,733,488	41.06	SAMN50434502
DF2212	20101747*I	SBG, Singapore	f	346,322,018	51.95	SAMN50434494
DF2213	20101747*H	SBG, Singapore	f	244,594,244	36.69	SAMN50434495
DF2214	20101747*G	SBG, Singapore	m	300,967,582	45.15	SAMN50434503
DF2215	20101747*F	SBG, Singapore	f	341,294,826	51.2	SAMN50434496
DF2216	20101747*E	SBG, Singapore	f	329,933,496	49.49	SAMN50434497
DF2217	20192592*J	SBG, Singapore	m	325,605,212	48.84	SAMN50434504
DF2218	20192592*K	SBG, Singapore	f	237,841,254	35.68	SAMN50434498
DF2219	20192592*H	SBG, Singapore	m	276,686,000	41.5	SAMN50434505
DF2220	JY-02809	SBG, Singapore	m	306,898,326	46.03	SAMN50434506
DF2222	20192592*G	SBG, Singapore	f	252,876,286	37.93	SAMN50434499
E001	NA	Eden proj. UK	f	330,333,564	49.55	SAMN50434500
E002	NA	Eden proj. UK	m	330,148,388	49.52	SAMN50434507

[1)] SBG = Singapore Botanic Gardens, Singapore

Table S6. Functional annotation of genes found within the Y- and X-SLR on chromosome 6. Gene name was attributed by BRAKER3.

**Haplotype 1 (Y-SLR)**				
Gene name	e-value	Bit-score	Description	PFAMs
g7204.t1	1.50E-20	90.1	-	-
g7205.t1	7.01E-24	95.5	-	-
g7206.t1	1.05E-22	100	Mitotic sister chromatid biorientation	-
g7207.t1	2.59E-06	53.9	-	-
g7208.t1	1.50E-20	90.1	-	-
g7209.t1	1.02E-22	96.7	-	-
g7210.t1	3.90E-53	167	-	-
g7211.t1	2.49E-89	267	-	-
g7212.t1	2.07E-49	179	Integrase core domain	Retrotrans_gag, rve
g7213.t1	5.11E-53	192	8-hydroxy-dADP phosphatase activity	Retrotrans_gag
g7214.t1	4.34E-97	283	Destroys radicals which are normally produced within the cells and which are toxic to biological systems	Sod_Cu
g7215.t1	3.36E-57	184	-	-
g7216.t1	6.58E-13	70.1	Calmodulin-binding transcription activator	Ank_2,Ank_4,CG-1,IQ,TIG
g7217.t1	2.38E-242	721	DNAJ heat shock N-terminal domain-containing protein	DUF3444,DnaJ
g7218.t1	3.17E-59	217	DNAJ heat shock N-terminal domain-containing protein	DUF3444,DnaJ
g7219.t1	3.45E-214	661	BRCT domain-containing DNA repair protein	RTT107_BRCT_5
g7220.t1	1.66E-270	754	Glycosyltransferase	Exostosin
g7220.t2	1.15E-247	693	Glycosyltransferase	Exostosin
g7221.t1	3.13E-305	848	Carotenoid cleavage dioxygenase 7	RPE65
g7224.t1	1.90E-35	131	Gag-polypeptide of LTR copia-type	Retrotran_gag_2
g7225.t1	2.85E-70	239	DNA RNA polymerases superfamily protein	RVP_2,RVT_1, Retrotrans_gag, rve, zf-CCHC
g7226.t1	6.17E-57	204	Retroviral aspartyl protease	RVP_2,RVT_1, Retrotrans_gag, zf-CCHC
g7227.t1	2.97E-07	54.3	DNA RNA polymerases superfamily protein	RVP_2,RVT_1, Retrotrans_gag, rve, zf-CCHC
g7228.t1	1.62E-51	176	Gag-polypeptide of LTR copia-type	Retrotran_gag_2, gag_pre-integrs, zf-CCHC
g7229.t1	1.22E-39	152	strictosidine synthase activity	Asp_protease_2, RVT_1, Retrotrans_gag, rve
g7230.t1	-	-	-	-
g7231.t1	1.01E-51	182	E3 ubiquitin-protein ligase	zf-RING_2
g7232.t1	1.00E-72	237	KH domain	KH_6

g7233.t1	4.16E-87	261	ADP-ribosylation factor GTPase-activating protein	C2
g7233.t2	2.67E-84	253	ADP-ribosylation factor GTPase-activating protein	C2
g7234.t1	2.77E-35	120	-	-
g7235.t1	7.47E-53	201	DCD	Dev_Cell_Death
**Haplotype 2 (X-SLR)**				
g7491.t1	4.34E-97	283	Destroys radicals which are normally produced within the cells and which are toxic to biological systems	Sod_Cu
g7492.t1	3.36E-57	184	-	-
g7493.t1	2.69E-244	726	DNAJ heat shock N-terminal domain-containing protein	DUF3444, DnaJ
g7494.t1	2.72E-60	215	Domain of unknown function (DUF3444)	DUF3444, DnaJ
g7495.t1	1.89E-213	659	BRCT domain-containing DNA repair protein	RTT107_BRCT_5
g7496.t1	5.91E-87	261	ADP-ribosylation factor GTPase-activating protein	C2
g7496.t2	2.67E-84	253	ADP-ribosylation factor GTPase-activating protein	C2
g7497.t1	5.60E-35	120	-	-
g7498.t1	3.60E-52	199	DCD	Dev_Cell_Death

Table S7. Primers developed in this study. The sex-specific primer pair is in bold. _F and _R denote the forward and reverse primers, respectively. The annealing temperature is the temperature used in the PCR protocol.

No.	Name	Sequence	Length	GC (%)	Annealing temp. (°C)
7609	7609_F	TCACCCTGAGCCTTTGAG	18	55,5	58
	7609_R	CAGCACATCACAAAACCCC	18	52,6	58
7606	7606_F	CCTCATCATTTTCCACCTCAAC	22	45,5	55
	7606_R	GCCTCATTTGATAGAGAAGACAC	23	43,5	55
7607	7607_F	GAGCTTAGGTCACTATCTCCC	21	52,4	55
	7607_R	CACAAAACCCCCATTCTTCAC	21	47,6	55
7613	7613_F	GCATTCGTAACACACCACTTTC	22	45,5	58
	7613_R	GCATTACCAAGCCTCCATTC	20	50	58
7619	7619_F	CTGAGCCTTTGAGTTTGCTG	20	50	58
	7619_R	CACAAAACCCCCATTCTTCAC	21	47,6	58
7624	7624_F	GTGTCAGCAGTGATCCATATAG	22	45,5	55
	7624_R	CAACCATGTGATCGGGGAG	19	57,9	55
7631	7631_F	TCATCATCATCTTCAAACGCC	21	42,9	58
	7631_R	CTTTCATCCCCACCACAAC	19	52,6	58
7634	7634_F	ATCTGTGAGCAACAACTTCC	20	45	55
	7634_R	TGTCTATAAACTGACCAAAGCC	22	40,9	55
7638	7638_F	GCCCCATTCAAAACCATATCAC	22	45,5	58
	7638_R	CATCTTCCACCTCAACCAAC	20	50	58
7640	7640_F	ACCTCCTTGGCACTTTCAC	19	52,6	58
	7640_R	CACATTACAAAACCCCCTTTCTTC	24	41,7	58
7642	7642_F	GGCATCAGAAATGATCAACAAC	22	40,9	55
	7642_R	CATAGCATCTTCACTCTCACTTAC	24	41,7	55
7649	7649_F	AATGAATGTAAGTGTTGGGAGG	22	40,9	55
	7649_R	TTGAGAGAACAAGGATTGGAAG	22	40,9	55
7651	7651_F	CTGCTATGTGCTCTTGTTCC	20	50	55
	7651_R	TCTTCTTGCCTTCTTTTTCCTG	22	40,9	55
7662	7662_F	TCCCATTACCTTCCTTTCCC	20	50	58
	7662_R	AGCCCATTTCATTGCTGTC	19	47,4	58
7663	7663_F	CATATCCTACCTTATCCTCATCCC	24	45,8	55
	7663_R	CCCAAGAGACCTAAGCAAAC	20	50	55
7668	7668_F	ATCTTTCTTCAATCCATCACCC	22	40,9	55
	7668_R	GTTACCTCATCGTCATCGTC	20	50	55
7671	7671_F	TGAGAAGAACAAGACATGGAAG	22	40,9	55
	7671_R	CAGATGAAGAGCAAGCGG	18	55,6	55

7618	7618_F	CCTTCATCCCCACCATGTC	19	57,9	58
	7618_R	TCCATATAGCTCTTCCAAGCTC	22	45,5	58
7641	7641_F	AACTCAAATCACTTTCACCCC	21	42,9	55
	7641_R	ACTGCAATCGAAGAAGCAC	19	47,4	55
7652	7652_F	CCCAAACTTTCTTAGCACTTTC	22	40,9	55
	7652_R	ACATCACAAAACCCCCATTC	20	45	55
7216L	7216L_F	TTGAGGATGCTGTCTTCGTG	20	50	57
	7216L_R	ATGGTCCCCAAAAACCTCAC	20	50	57
**7216M2**	**7216M2_F**	**CTTGATGCGTTGATTGGCTG**	20	50	57
	**7216M2_R**	**AAACGGGCCTGTATAACCAC**	20	50	57
7216M1	7216M1_F	GCCAACAGTGACAATTGCAG	20	50	57
	7216M1_R	ATGGTCCCCAAAAACCTCAC	20	50	57
7216S	7216S_F	GAGGATGCTGTCTTCGTGG	19	57.8	57
	7216S_R	CGACGCAGAGATGAAAGAGC	20	55	57
7171t1_1	7171t1_1_F	AACGGTATCCAACGTGTCAG	20	50	57
	7171t1_1_R	TTTCGGCGAGGTATGTGAAG	20	50	57
7172t1_1	7172t1_1_F	AGGATAGGTCTCACACGTCC	20	55	57
	7172t1_1_R	GAAGACTTGCGCAAGAACAC	20	50	57
7172_2	7172_2_F	GCCACGTTCATCTTGACAAC	20	50	57
	7172_2_R	CTCCCTCTTTCATGAGCCTG	20	55	57
7172_3	7172_3_F	TGCCACTGCAGACATTGTAG	20	50	57
	7172_3_R	ACTTCTGACACTGGCACATG	20	50	57

Table S8. PCR protocol for developed primers. The annealing temperature for each primer corresponds to the temperature reported in S5.

Contents	Volume per sample (PCR) (μL)
Water, nuclease-free	41.25
10X Dream Taq Buffer	5
dNTP Mix, 10mM	1
Dream Taq Polymerase	0.25
Total Vol without primers	47.5
Forward Primer	0.25
Reverse Primer	0.25
Total Volume without DNA	48
Template DNA	2
Total Volume	50

Step	Temperature °C	Time	No of cycles
Initial denaturation	95	3 min	1
Denaturation	95	30s	40
Annealing	*	30s	
Extension	72	1 min	
Final Extension	72	5 -15 min	1

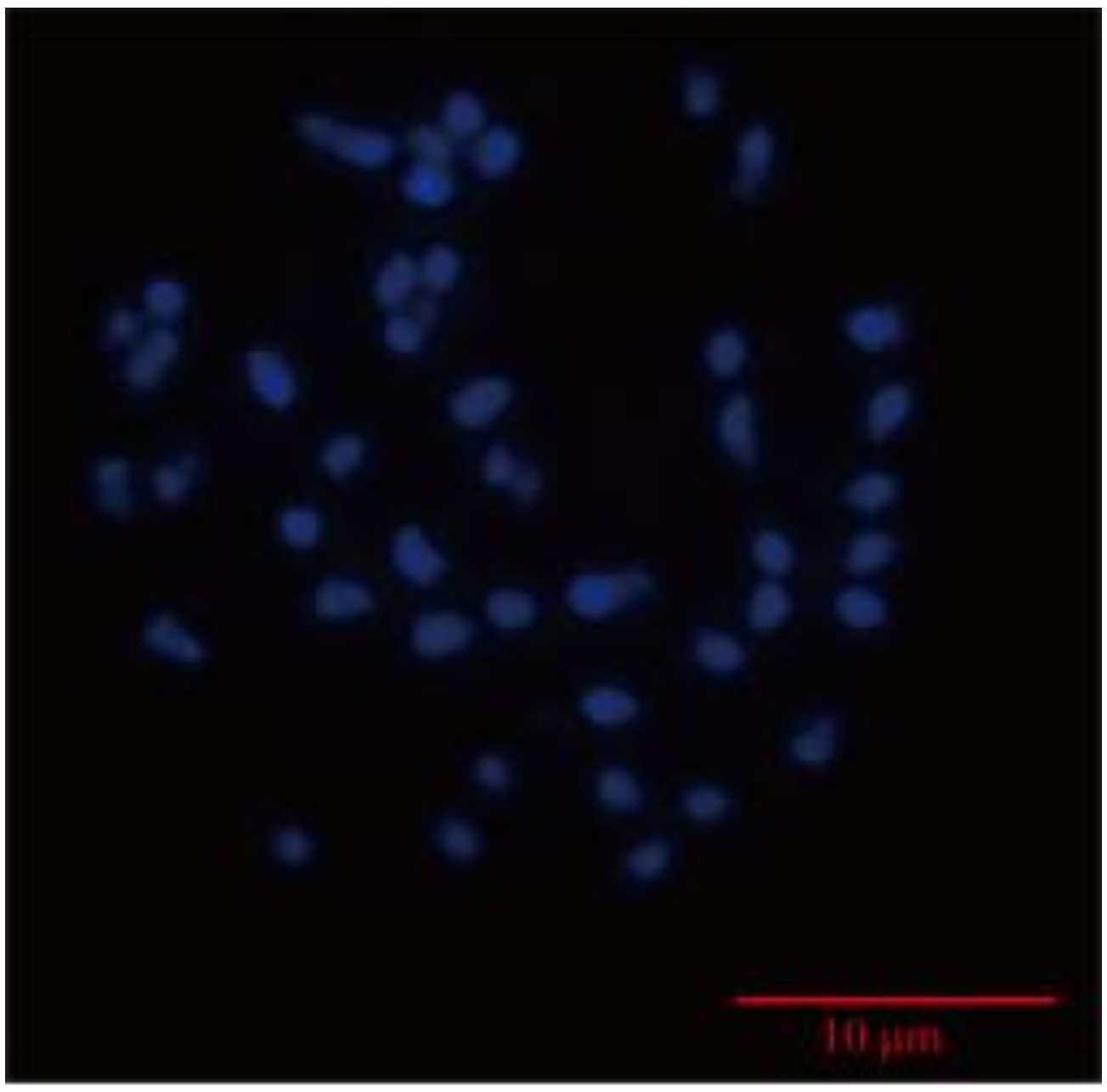

Figure S1. Mitotic chromosomes from root tip of *M. fragrans*. The scale bar corresponds to 10 μm.

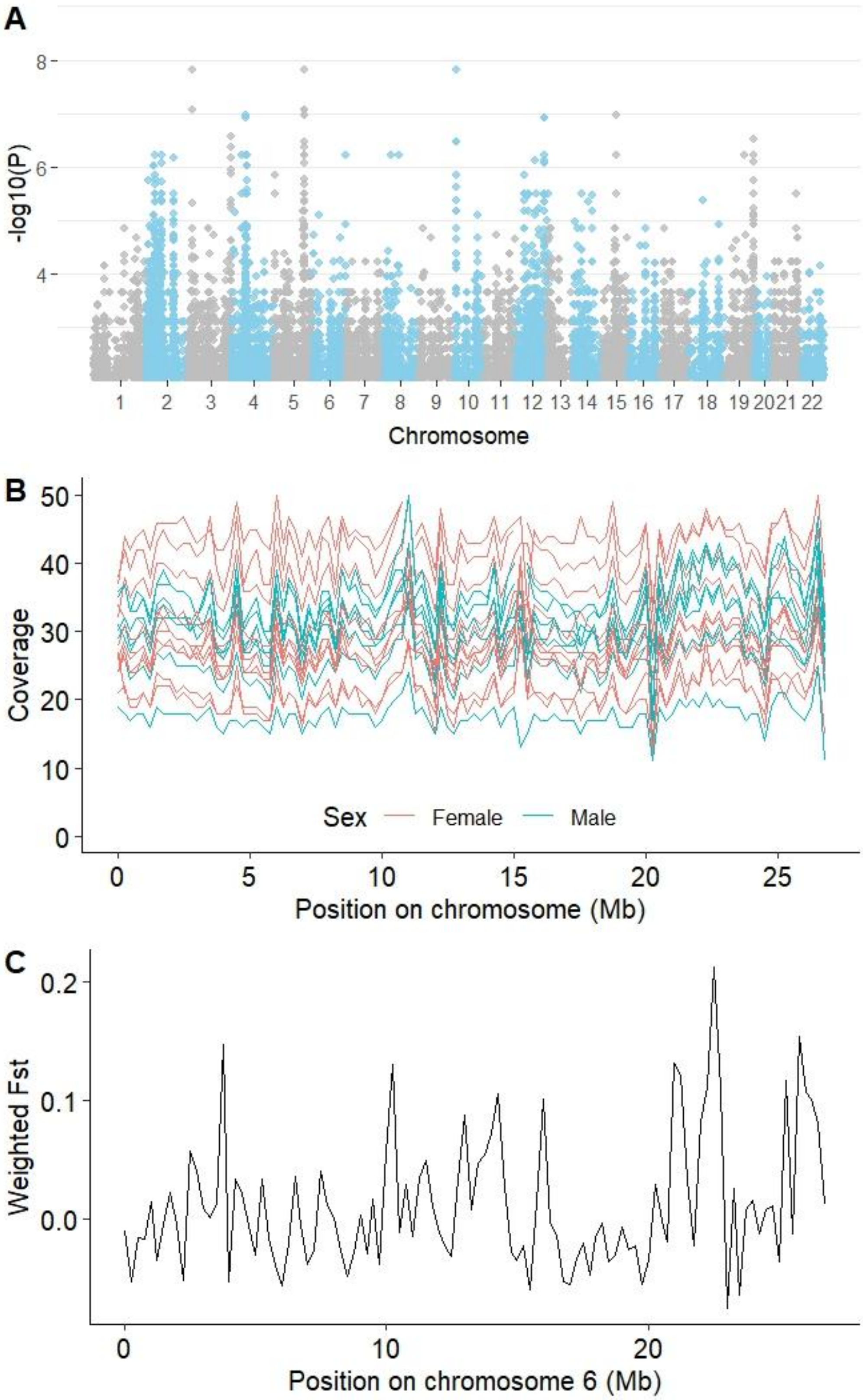

Figure S2. Analyses used to identify candidate SLR. **A)** Genome-wide association study on hap2 using sex as the phenotypical trait. **B)** Mean coverage on chromosome 6 of all sampled individuals mapped to the hap2 genome using 250 kb windows. **C)** F_{st} values across chromosome 6 of hap2 using a 500 kb sliding window.

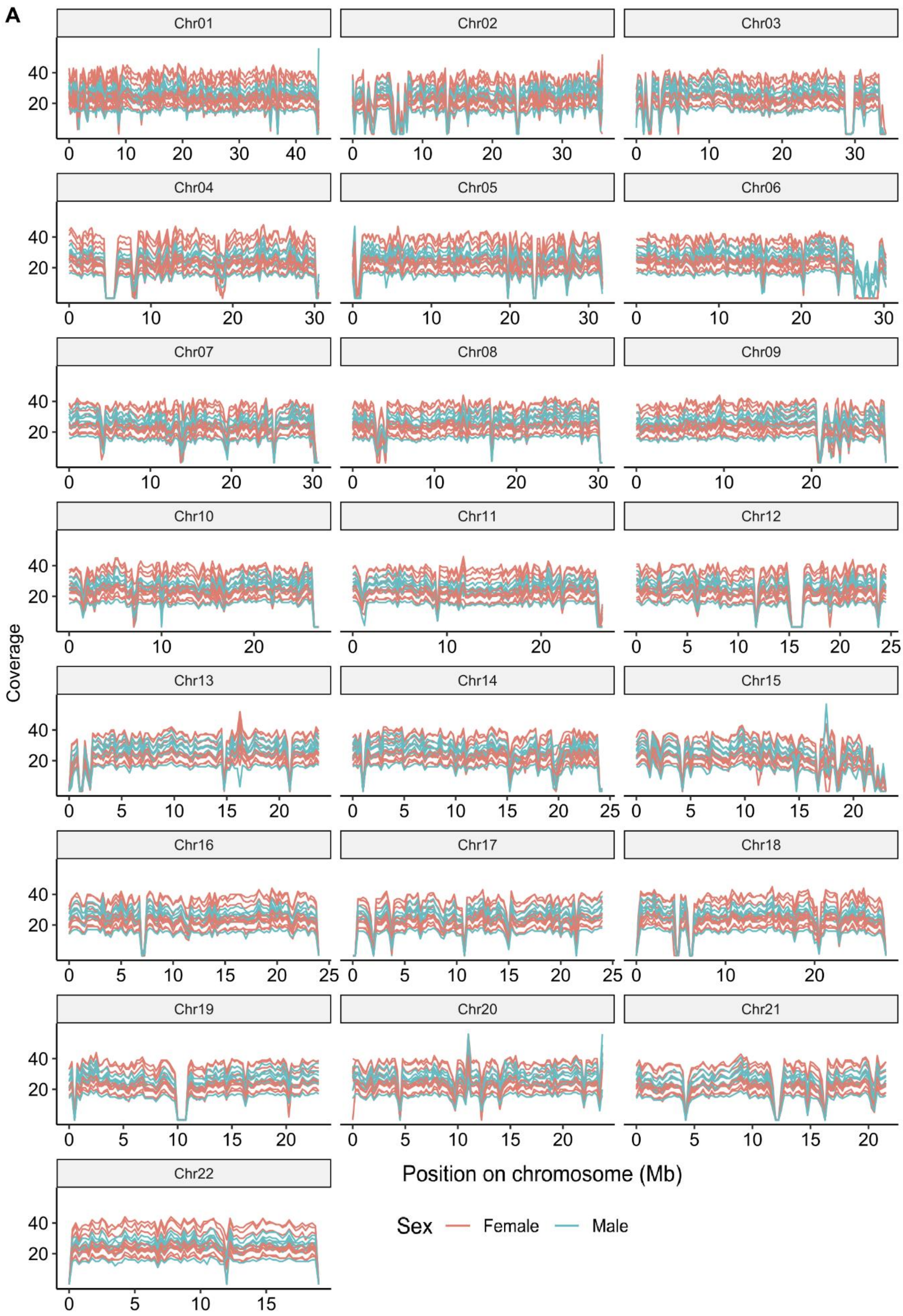
A
Chr01
Chr02
Chr03
Chr04
Chr05
Chr06
Chr07
Chr08
Chr09
Chr10
Chr11
Chr12
Chr13
Chr14
Chr15
Chr16
Chr17
Chr18
Chr19
Chr20
Chr21
Chr22
Coverage
Position on chromosome (Mb)
Sex
Female
Male

56

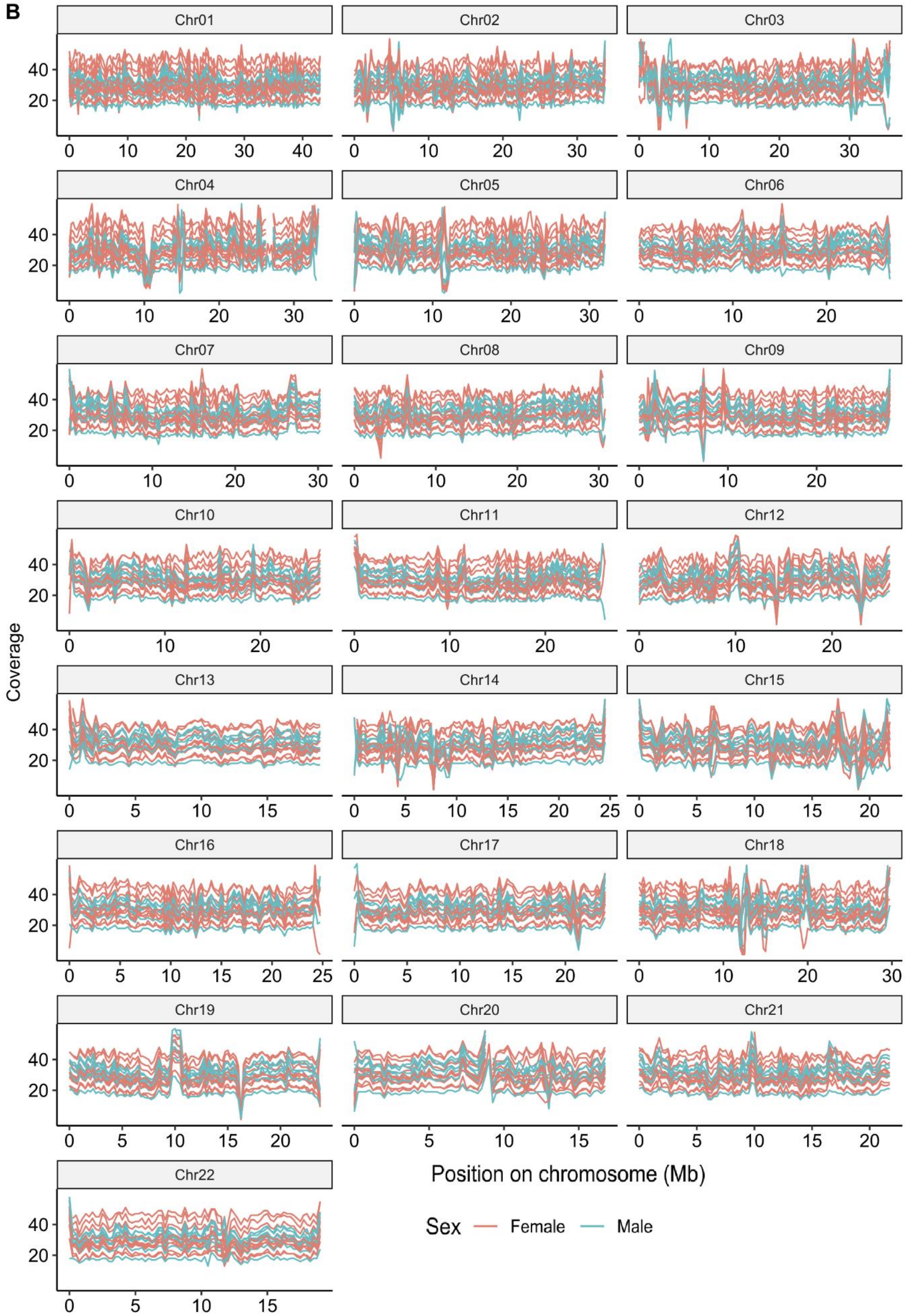

Figure S3. Genome-wide sequence coverage in 250kb windows for hap1 (A) and hap2 (B) using Illumina sequence data from 7 male (blue) and 11 female (red) samples.

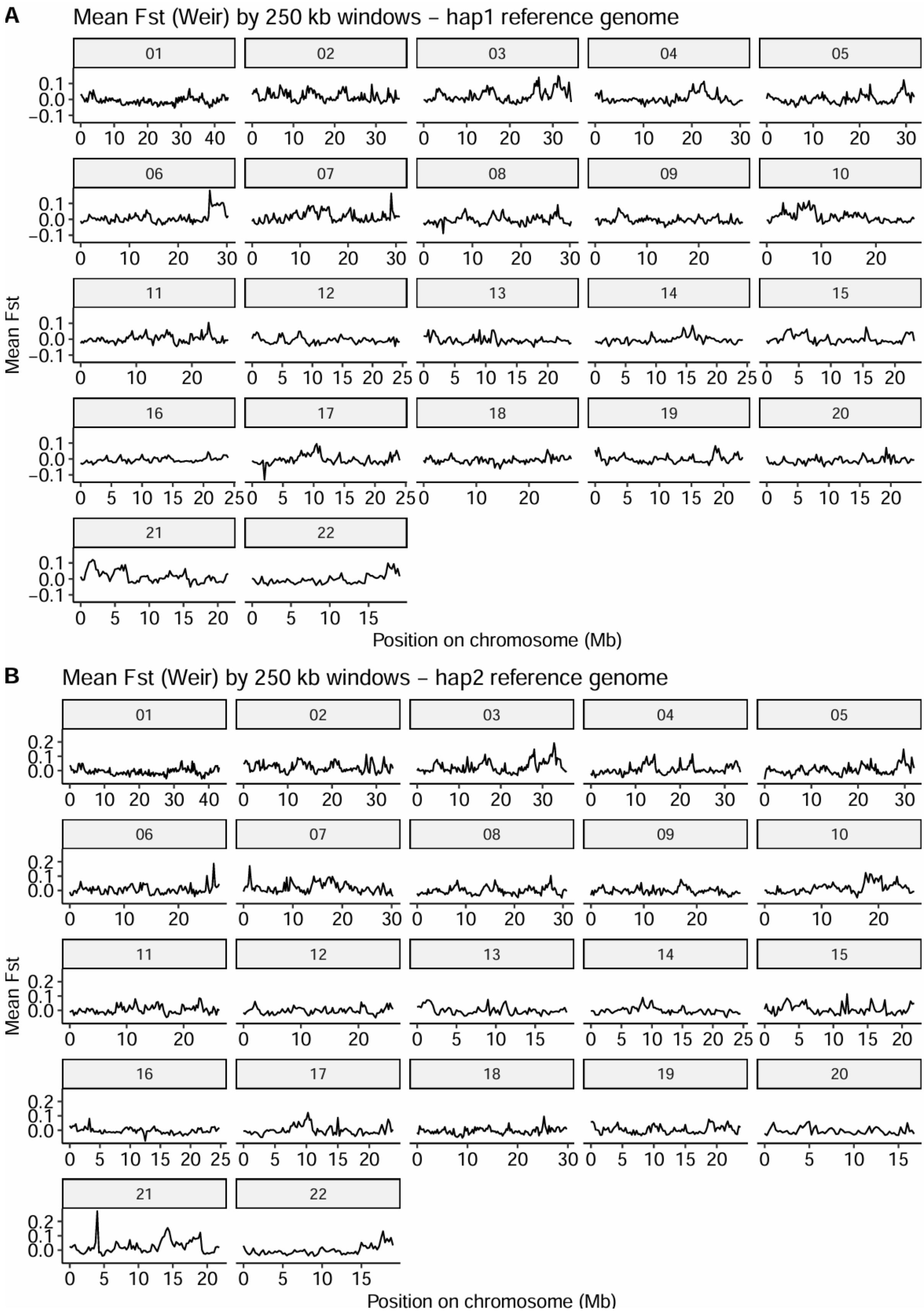

Figure S4. Genome-wide F_{st} in 250kb windows between males and females using hap1 (A) or hap2 (B) as the reference sequence.

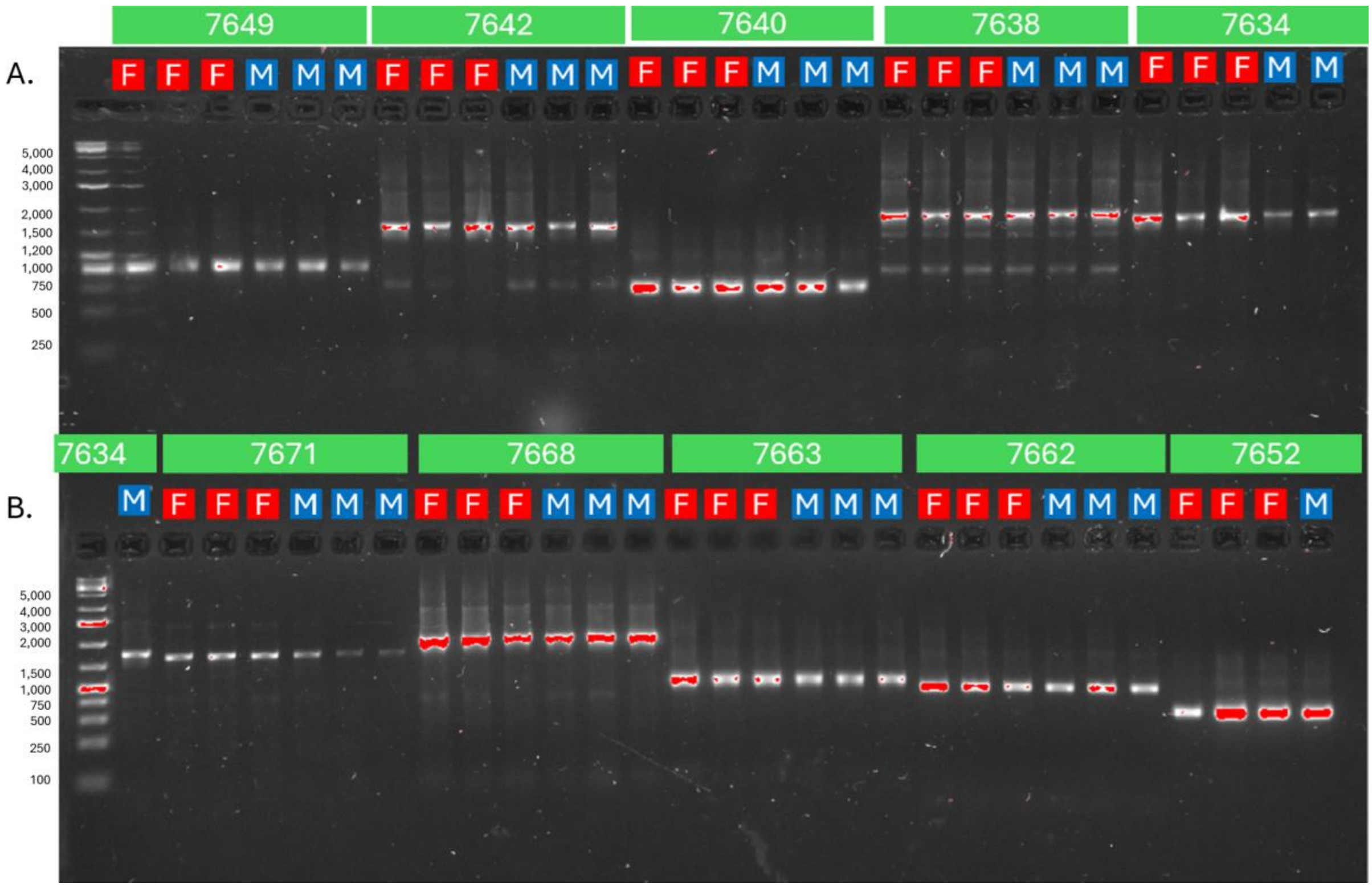

A.
7649
7642
7640
7638
7634
F F F M M M F F F M M M F F F M M M F F F M M M F F F M M
5,000
4,000
3,000
2,000
1,500
1,200
1,000
750
500
250
B.
7634
7671
7668
7663
7662
7652
M F F F M M M F F F M M M F F F M M M F F F M M M F F F M
5,000
4,000
3,000
2,000
1,500
1,000
750
500
250
100

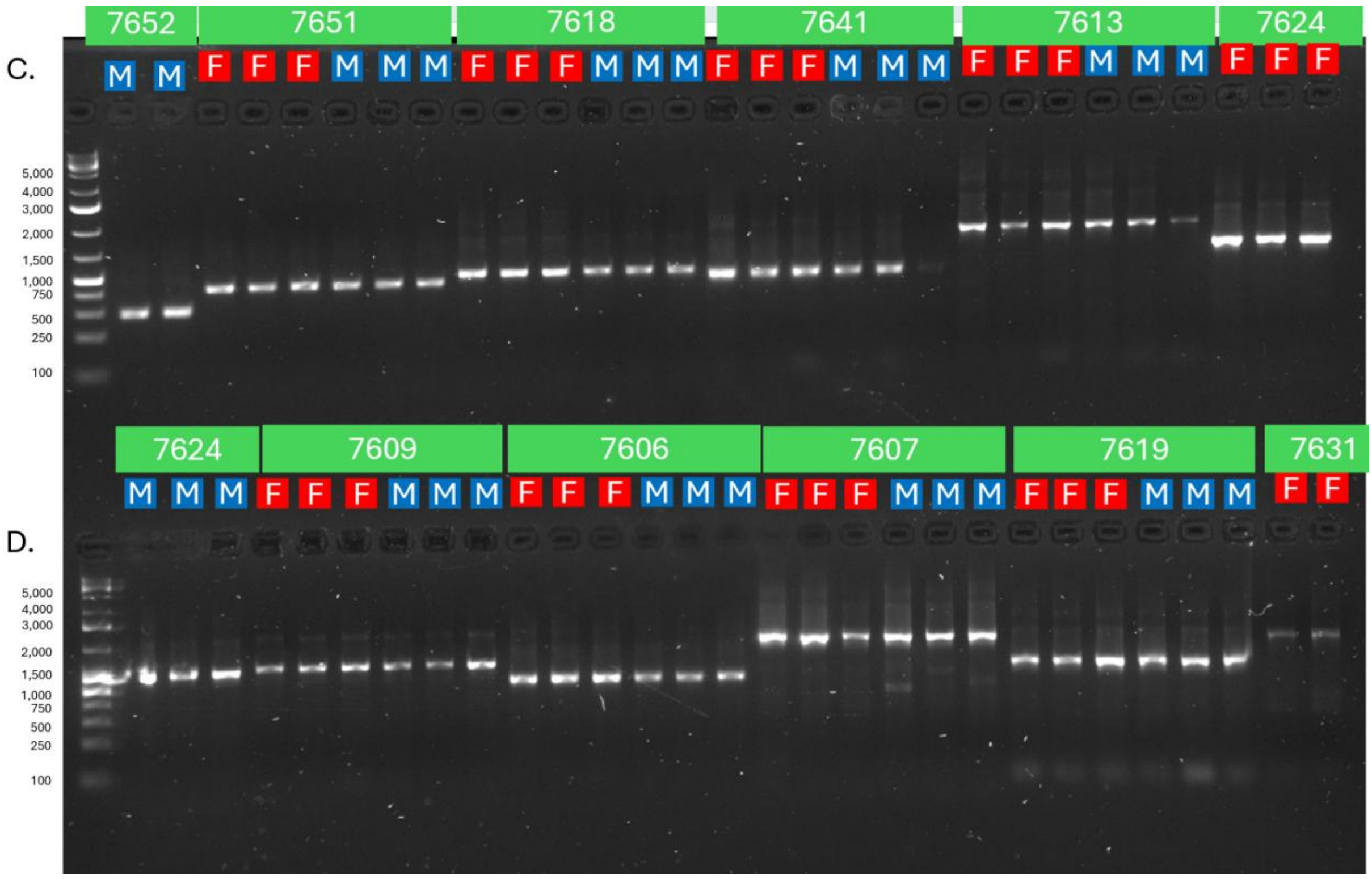

C.
7652
7651
7618
7641
7613
7624
M M F F F M M M F F F M M M F F F M M M F F F M M M F F F
5,000
4,000
3,000
2,000
1,500
1,000
750
500
250
100
D.
7624
7609
7606
7607
7619
7631
M M M F F F M M M F F F M M M F F F M M M F F F M M M F F
5,000
4,000
3,000
2,000
1,500
1,000
750
500
250
100

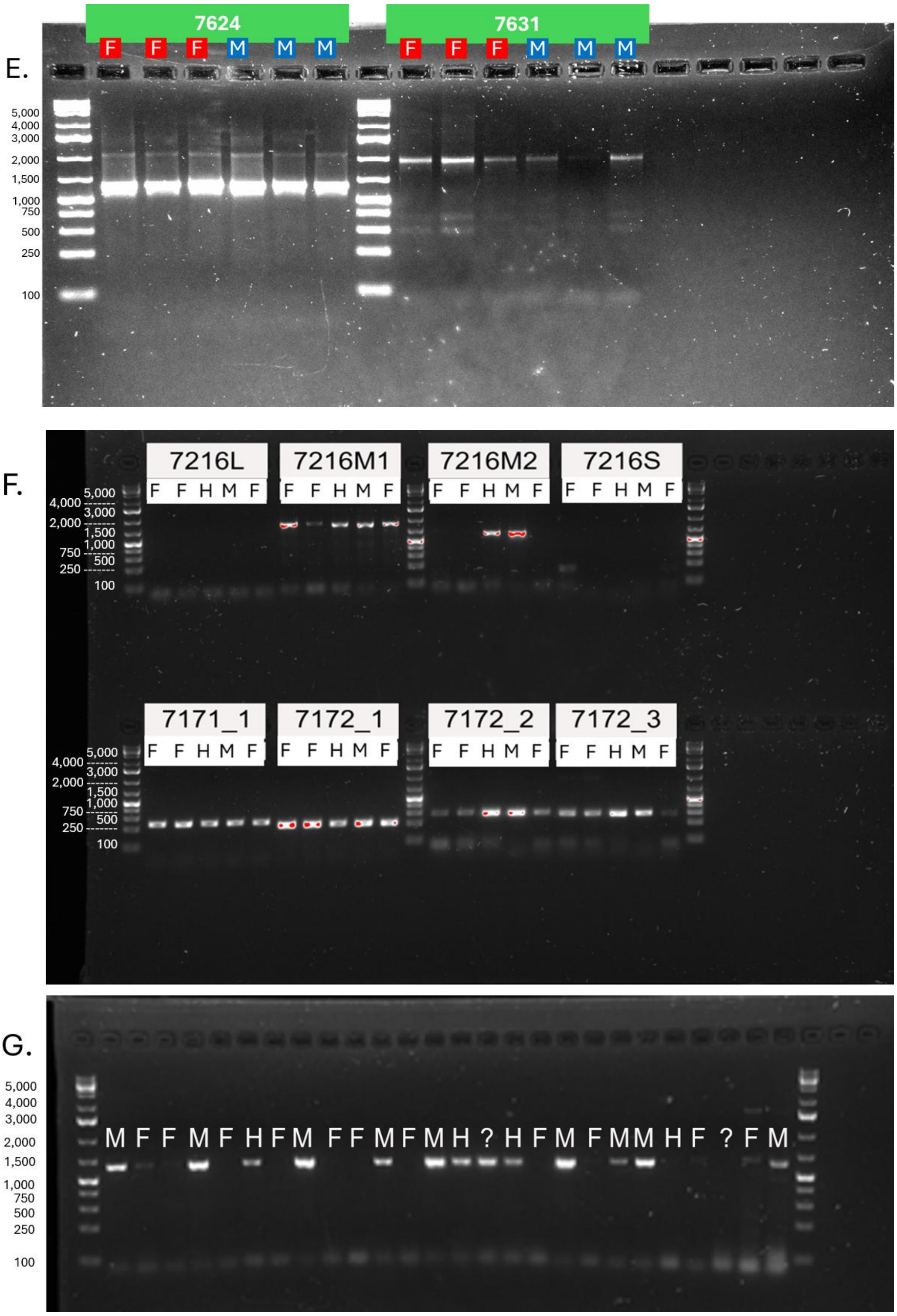

Figure S5. Electrophoresis gels of the developed primers with 1 kb ladders used on all gels. A-E. 20 developed markers that showed no sex specificity. F. Gel with eight markers, where one (7216_M2) showed sex specificity. G. Electrophoresis gel of PCR products from 26 individuals amplified with the sex-specific primer pair Mf_7216M2. “M”, “F” and “H” denote male, female and hermaphrodite (monoecious) individuals. For two individuals sex was not known (marked with “?”). Promega 1kb ladder was used as the molecular marker on the gels. The numbers to the left or each gel show the size of the bands in the ladder in nucleotides.

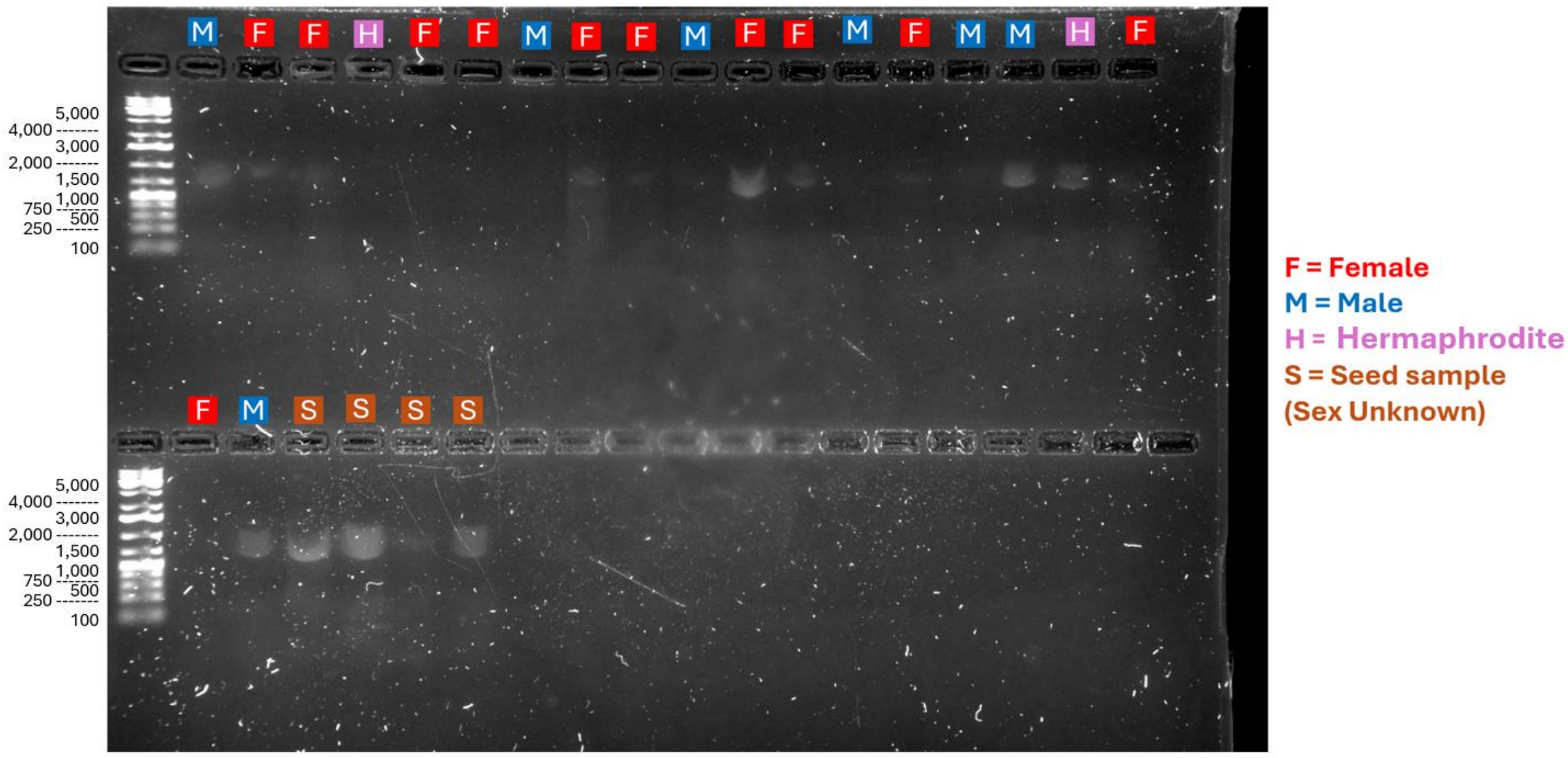

Figure S6. Electrophoresis gel of the previously published primers (OPE11) and Promega 1 kb ladder. The numbers to the left or each gel show the size of the bands in the ladder in nucleotides.
