# Supplementary material for: A haplotype-resolved nutmeg genome reveals XY sex chromosome evolution and enables molecular sexing
Source: Commun Biol. 2026 May 28;9:1132. doi: 10.1038/s42003-026-10355-0 (PMC13500479; doi:10.1038/s42003-026-10355-0)
Supplement: Supplementary file 2 [file 42003_2026_10355_MOESM2_ESM.pdf]

nature portfolio

Corresponding author(s): Ray Ming and Dmitry Filatov

Last updated by author(s): 21/04/2026

Reporting Summary

Nature Portfolio wishes to improve the reproducibility of the work that we publish. This form provides structure for consistency and transparency in reporting. For further information on Nature Portfolio policies, see our Editorial Policies and the Editorial Policy Checklist.

Statistics

For all statistical analyses, confirm that the following items are present in the figure legend, table legend, main text, or Methods section.

n/a	Confirmed	
☐	☒	The exact sample size (n) for each experimental group/condition, given as a discrete number and unit of measurement
☐	☒	A statement on whether measurements were taken from distinct samples or whether the same sample was measured repeatedly
☐	☒	The statistical test(s) used AND whether they are one- or two-sided<br>*Only common tests should be described solely by name; describe more complex techniques in the Methods section.*
☒	☐	A description of all covariates tested
☐	☒	A description of any assumptions or corrections, such as tests of normality and adjustment for multiple comparisons
☐	☒	A full description of the statistical parameters including central tendency (e.g. means) or other basic estimates (e.g. regression coefficient) AND variation (e.g. standard deviation) or associated estimates of uncertainty (e.g. confidence intervals)
☐	☒	For null hypothesis testing, the test statistic (e.g. F, t, r) with confidence intervals, effect sizes, degrees of freedom and P value noted<br>*Give P values as exact values whenever suitable.*
☒	☐	For Bayesian analysis, information on the choice of priors and Markov chain Monte Carlo settings
☒	☐	For hierarchical and complex designs, identification of the appropriate level for tests and full reporting of outcomes
☒	☐	Estimates of effect sizes (e.g. Cohen's d, Pearson's r), indicating how they were calculated

Our web collection on statistics for biologists contains articles on many of the points above.

Software and code

Policy information about availability of computer code

Data collection	Data collection only included DNA sequencing that was done by genomic facility.
Data analysis	Data analysis was done with publicly available software packages described in the methods, such as Trimmomatic (to trim sequence reads), bwa to map reads to reference, samtoools to process bam files and generate vcf files, proseq4 to process and analyse vcf files.

For manuscripts utilizing custom algorithms or software that are central to the research but not yet described in published literature, software must be made available to editors and reviewers. We strongly encourage code deposition in a community repository (e.g. GitHub). See the Nature Portfolio guidelines for submitting code & software for further information.

Data

Policy information about availability of data

All manuscripts must include a data availability statement. This statement should provide the following information, where applicable:

- Accession codes, unique identifiers, or web links for publicly available datasets
- A description of any restrictions on data availability
- For clinical datasets or third party data, please ensure that the statement adheres to our policy

Genome and transcriptome sequence data generated in this project are available from NCBI (https://www.ncbi.nlm.nih.gov/) under project number PRJNA1301071. The genome assemblies are available from Genome Warehouse repository (https://ngdc.cncb.ac.cn/gwh/) under project number PRJCA063189.

Research involving human participants, their data, or biological material

Policy information about studies with human participants or human data. See also policy information about sex, gender (identity/presentation), and sexual orientation and race, ethnicity and racism.

Reporting on sex and gender	no human participants used - this study used plants, not humans
Reporting on race, ethnicity, or other socially relevant groupings	no human participants used - this study used plants, not humans
Population characteristics	no human participants used - this study used plants, not humans
Recruitment	no human participants used - this study used plants, not humans
Ethics oversight	no human participants used - this study used plants, not humans

Note that full information on the approval of the study protocol must also be provided in the manuscript.

Field-specific reporting

Please select the one below that is the best fit for your research. If you are not sure, read the appropriate sections before making your selection.

☐ Life sciences ☐ Behavioural & social sciences ☒ Ecological, evolutionary & environmental sciences

For a reference copy of the document with all sections, see nature.com/documents/nr-reporting-summary-flat.pdf

Ecological, evolutionary & environmental sciences study design

All studies must disclose on these points even when the disclosure is negative.

Study description	We sequenced the genome of nutmeg and identified its sex-linked region (SLR). To identify SLR we compared the genetic composition of male and female nutmeg plants and looked for the regions that have significant differences between males and females using several independent approaches.
Research sample	All male and female plants we used came from botanic gardens (see table S5 in suppl. material)
Sampling strategy	We used 11 female and 7 male plants to identify the sex-linked region. Based on the previous studies, at least 4 samples of each sex are needed to reliably identify sex-linked region in a sequenced genome. Thus, our sample included two to three times the minimal number of individuals needed for identification of the sex-linked region.
Data collection	All data we used are DNA sequence data, which we made publicly available. That data were "collected" by sequencing machines.
Timing and spatial scale	not applicable
Data exclusions	no data were excluded from the analyses
Reproducibility	Identification of sex-linked region was conducted by four different approaches, which all consistently pointed to the same genomic region.
Randomization	The only allocation of samples was to male and female groups, which was done based on the phenotype of flowers (pistilate versus staminate).
Blinding	We started the analysis without any prior knowledge of where the sex-linked region is in the genome, which is the only "blinding" applicable to this study. Using independent approaches to find the sex-linked region, we consistently identify the same genomic region.

Did the study involve field work? ☐ Yes ☒ No

Reporting for specific materials, systems and methods

We require information from authors about some types of materials, experimental systems and methods used in many studies. Here, indicate whether each material, system or method listed is relevant to your study. If you are not sure if a list item applies to your research, read the appropriate section before selecting a response.

Materials & experimental systems

n/a	Involved in the study
☒	☐ Antibodies
☒	☐ Eukaryotic cell lines
☒	☐ Palaeontology and archaeology
☒	☐ Animals and other organisms
☒	☐ Clinical data
☒	☐ Dual use research of concern
☐	☒ Plants

Methods

n/a	Involved in the study
☒	☐ ChIP-seq
☐	☒ Flow cytometry
☒	☐ MRI-based neuroimaging

Dual use research of concern

Policy information about dual use research of concern

Hazards

Could the accidental, deliberate or reckless misuse of agents or technologies generated in the work, or the application of information presented in the manuscript, pose a threat to:

No	Yes
☒	☐ Public health
☒	☐ National security
☒	☐ Crops and/or livestock
☒	☐ Ecosystems
☒	☐ Any other significant area

Experiments of concern

Does the work involve any of these experiments of concern:

No	Yes
☒	☐ Demonstrate how to render a vaccine ineffective
☒	☐ Confer resistance to therapeutically useful antibiotics or antiviral agents
☒	☐ Enhance the virulence of a pathogen or render a nonpathogen virulent
☒	☐ Increase transmissibility of a pathogen
☒	☐ Alter the host range of a pathogen
☒	☐ Enable evasion of diagnostic/detection modalities
☒	☐ Enable the weaponization of a biological agent or toxin
☒	☐ Any other potentially harmful combination of experiments and agents

Plants

Seed stocks	No seeds were used in this study
Novel plant genotypes	No novel genotypes were created - we did not do any genetic modification, nor genetic crosses
Authentication	No authentication for the plants was necessary because the plants we used came already authenticated from various botanic gardens.

Flow Cytometry

Plots

Confirm that:

- [x] The axis labels state the marker and fluorochrome used (e.g. CD4-FITC).
- [x] The axis scales are clearly visible. Include numbers along axes only for bottom left plot of group (a 'group' is an analysis of identical markers).
- [ ] All plots are contour plots with outliers or pseudocolor plots.
- [ ] A numerical value for number of cells or percentage (with statistics) is provided.

Methodology

Sample preparation	Fresh tissue was chopped in ice-cold Mg^{2+} buffer, filtered through a 40-µm nylon mesh, and nuclei were stained with propidium iodide (50 µg/mL) in the presence of RNase A. Stained nuclei were analysed on flow cytometer with 488-nm excitation. Genome size was calculated from the ratio of mean fluorescence intensities of the sample and the internal standard, multiplied by the known genome size of the standard.
Instrument	BD FACScalibur flow cytometer
Software	Data were processed with ModFit LT v3.0
Cell population abundance	At least 10,000 nuclei per sample were analysed
Gating strategy	Gating strategy is shown on figure S7 in the manuscript. First, debris was excluded by gating on FSC-H/SSC-H dot plots (left panel, fig.S7). Next, nuclei were gated based on PI-H/SSC-H characteristics (middle panel, figS7). Finally, DNA content histograms (PI-H vs. Counts) were generated to display the distribution of cell cycle phases (right panel, fig.S7).

- [x] Tick this box to confirm that a figure exemplifying the gating strategy is provided in the Supplementary Information.
